# TIE2-positive cells in the nucleus pulposus with a purpose: the who, what and why

**DOI:** 10.1186/s12929-026-01220-7

**Published:** 2026-03-02

**Authors:** Jordy Schol, Luca Ambrosio, Clara Ruiz-Fernandez, Leon Schlagenhof, Chantal Voskamp, Lisanne T. Laagland, Erika Matsushita, Hazuki Soma, Takayuki Warita, Gianluca Vadalà, Marianna A. Tryfonidou, Benjamin Gantenbein, Daisuke Sakai

**Affiliations:** 1 Department of Orthopaedic Surgery, Tokai University School of Medicine, 143 Shimokasuya, Isehara, 259-1193 Japan; 2 Center for Musculoskeletal Innovative Research and Advancement (C-MiRA), Tokai University Graduate School, 143 Shimokasuya, Isehara, 259-1193 Japan; 3 Research Unit of Orthopaedic and Trauma Surgery, Departmental Faculty of Medicine and Surgery, Università Campus Bio-Medico di Roma, Via Alvaro del Portillo, 200, 00128 Rome, Italy; 4 Operative Research Unit of Orthopaedic and Trauma Surgery, Fondazione Policlinico Universitario Campus Bio-Medico, Via Alvaro del Portillo, 200, 00128 Rome, Italy; 5 Tissue Engineering for Orthopaedics & Mechanobiology, Bone & Joint Program, Department for BioMedical Research (DBMR), Faculty of Medicine, University of Bern, Murtenstrasse 35, CH-3008 Bern, Switzerland; 6 Graduate School for Cellular and Biomedical Sciences (GCB), University of Bern, Mittelstrasse 43, CH-3012 Bern, Switzerland; 7 Department of Orthopaedic Surgery and Traumatology, Inselspital, Bern University Hospital, Faculty of Medicine, University of Bern, Freiburgstrasse 3, CH-3010 Bern, Switzerland; 8 Department of Clinical Sciences, Faculty of Veterinary Medicine, Utrecht University, Yalelaan 108, 3584CM, Utrecht, the Netherlands; 9 Regenerative Medicine Center Utrecht, Yalelaan 108, 3584 CM Utrecht, the Netherlands; 10 TUNZ Pharma Corporation, 4-2-3 Hiranomachi, Chuo, Osaka, 541-0046 Japan

**Keywords:** Intervertebral disc, Progenitor cells, Notochord, Angiopoietins, Extracellular matrix, Cellular therapy, Inflammation, Intervertebral disc degeneration, Regenerative medicine

## Abstract

Once thought to be solely involved in vasculogenesis, tyrosine kinase with immunoglobulin-like and EGF-like domains 2 (TIE2) has emerged as a crucial marker of progenitor-like cells in the avascular nucleus pulposus (NP), a tissue with notoriously limited regenerative capacity. Recent evidence suggests that TIE2 + NP cells play a pivotal role in disc tissue homeostasis, influencing extracellular matrix maintenance, cellular renewal, and tissue integrity. However, despite the reported regenerative potential of TIE2 + NP cells, their precise function remains enigmatic. This review consolidates in vivo, in vitro, and transcriptomic studies to validate the presence of TIE2 in the NP as a progenitor cell marker. We unravel the complexity of TIE2 + NP cells across species, highlighting key regulatory mechanisms and interspecies variations (including mice, rats, dogs, cows, sheep, pigs, and humans) that may influence their relevance as clinical- and regenerative therapeutic targets. Yet, methodological inconsistencies across studies continue to obscure our understanding of the precise role of TIE2 in NP cell biology. At present, clinical care is limited to managing pain conservatively or resorting to spinal surgery in severe cases. Thus, there exists an urgent need for innovative regenerative strategies to combat disc degeneration and its associated pain and disability. A range of emerging approaches, including biomaterials, gene therapy, and cell-based therapeutics, are under investigation. Within this context, TIE2 + NP cells are of particular interest as potential therapeutic vectors: as for example candidate cells for transplantation, as populations to be stimulated by biologic interventions, or as building blocks in tissue engineering strategies. As progenitor-like cells, they hold the theoretical potential to provide a sustained source of functional NP cells for disc maintenance and repair. By identifying existing knowledge gaps and proposing future research directions, this review aims to clarify their role and accelerate progress toward unlocking their full therapeutic potential.

**Supplementary Information:**

The online version contains supplementary material available at 10.1186/s12929-026-01220-7.

## Introduction

### Disc degeneration, progenitor cell identity, and the role of cell surface markers

The intervertebral disc (IVD), specifically the nucleus pulposus (NP), is the largest avascular structure in the human body [1], presenting unique challenges for cell survival, nutrient diffusion, cell recruitment, and tissue maintenance [2–4]. Yet, paradoxically, tyrosine kinase with immunoglobulin-like and EGF-like domains 2 (TIE2), a receptor classically associated with angiogenesis and vasculogenesis [5–7], has been marked as a key regulator of the NP progenitor cell (NPPC) phenotype [8]. The stark contrast between the well-established role of TIE2 in vascular biology and its presence in the avascular NP underscores the further need to investigate its function in this unique environment. This would enhance our understanding of disc health as well as the potential implications of TIE2 in the more extensively studied fields of angiology and oncology.

The discovery of TIE2 dates back to the early 1990s: it was first identified as an endothelial cell-specific receptor involved in vascular development and angiogenesis [7, 9, 10]. Studies soon established its crucial role in vascular remodeling, vessel maturation, and hematopoietic stem cell maintenance [5, 7, 11], with further research linking TIE2 signaling to cancer progression [12]. Moreover, TIE2 has been implicated in lymphatic vessel formation, where it contributes to the remodeling and maturation of lymphatic vasculature through interactions with its ligands [13]. Given this well-defined function in vascular systems, the presence of TIE2 in the NP (a mechanically dynamic environment lacking both blood and lymphatic supply) was highly unexpected, prompting new questions regarding its regulatory mechanisms and potential role in cell renewal and extracellular matrix (ECM) maintenance [8]. Moreover, a decline in the number of TIE2 + NPPCs within the human NP tissue has been directly correlated with increasing age and advancing intervertebral disc degeneration (IDD) [8]. Since its identification, TIE2 + NP cells have been studied across multiple species, including humans, mice, rats, dogs, pigs, sheep, and cows, revealing key developmental and regulatory differences. TIE2 + NP cells are part of a tissue-specific progenitor population [14–16], involved in self-renewal, ECM maintenance, and tissue repair [8, 17]. However, their regulatory mechanisms remain poorly defined, raising questions about their activation, involved signaling pathways, and role in both IVD homeostasis and degeneration.

**Fig. 1:**
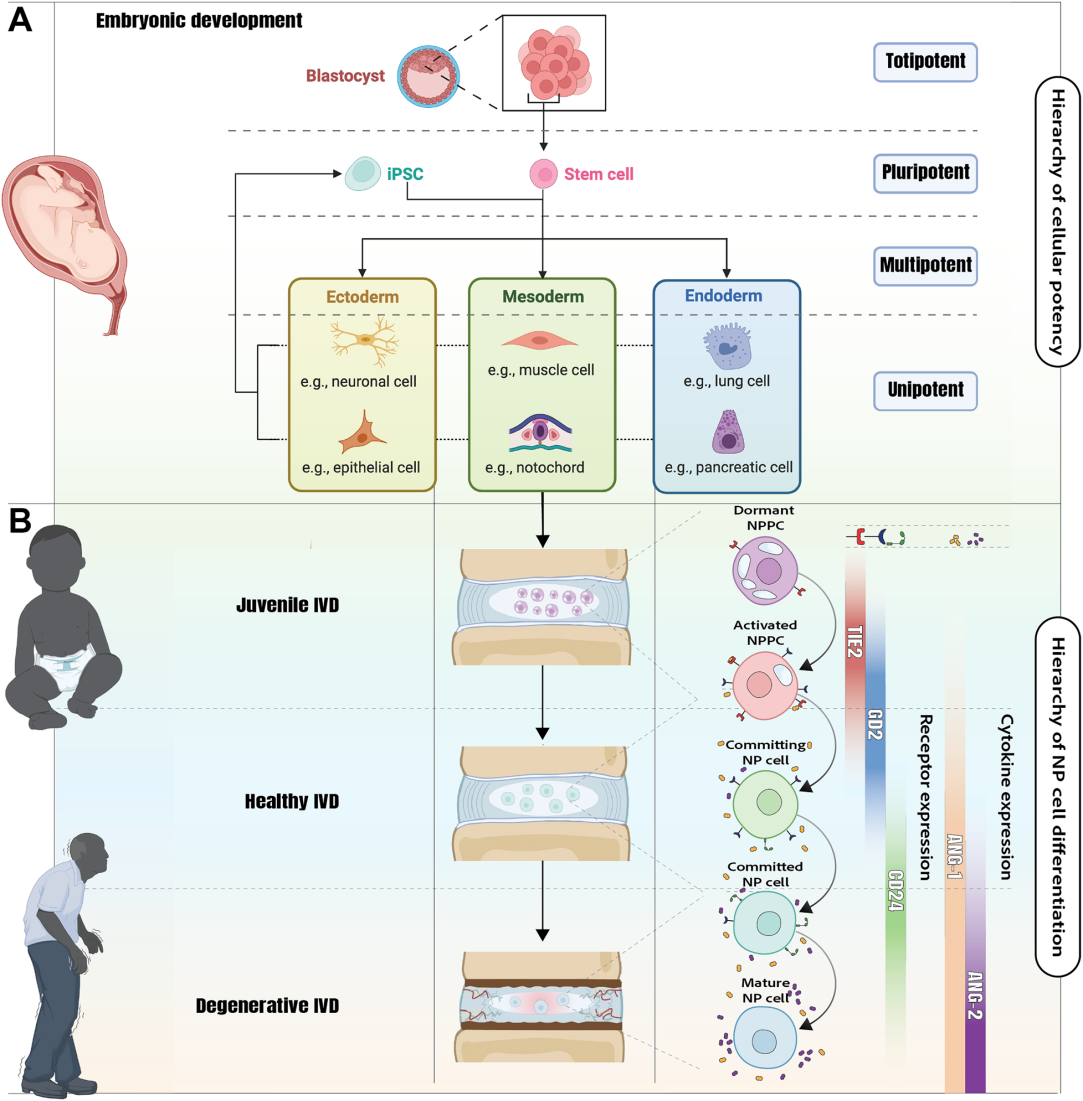
Developmental origin and TIE2-based hierarchy of NP cells across IVD aging. **A** Schematic overview of the differentiation potential of early embryonic stem cells, including totipotent, pluripotent, and multipotent lineages. Mesodermal differentiation gives rise to the notochord, the embryonic structure that develops into the NP of the IVD. **B** With aging, the NP shifts from a progenitor-rich juvenile tissue to a degenerative environment with reduced cell function. TIE2 + NPPCs form a functional hierarchy from dormant to mature states, with dynamic expression of TIE2, GD2, and CD24. The ANG/TIE2 signaling axis regulates NPPC activation and commitment, supporting their regenerative potential. Abbreviations: *ANG-1* Angiopoietin-1; *ANG-2* Angiopoietin-2; *CD24* Cluster of differentiation 24; *GD2* Disialoganglioside GD2; *iPSC* Induced pluripotent stem cell; *IVD* Intervertebral disc; *NP* Nucleus pulposus; *NPPC* Nucleus pulposus progenitor cell; *TIE2* Tyrosine kinase with immunoglobulin-like and EGF-like domains 2 receptor

Notably in disc homeostasis, a few species stand out due to the predominant cell types present in the NP [18, 19]. Notochordal cells, recognizable by their large cytoplasmic vacuoles, form the primary NP cell population during early development, which play a key role in ECM production and disc maintenance [19] (Fig. 1). However, in some species, including humans and chondrodystrophic dogs, sheep, goats and cows, these notochordal cells are replaced by smaller, non-vacuolated fibrocartilaginous NP cells rapidly after birth, resulting in changes in the composition of the NPECM [18–20]. This transition coincides with an increased susceptibility to early-onset IDD and discogenic low back pain (LBP) [18]. While this correlation is intriguing, the role of TIE2 + NPPCs in this differentiation process remains unclear, presenting a compelling area for further investigation. Especially as reports have highlighted the link between the progression of IDD and loss of TIE2 + NP cell populations within the disc (Fig. 1B) [8, 17]. Examining the presence and role of TIE2 in animal models that retain notochordal cells (such as rats, mice, pigs, rabbits, and non-chondrodystrophic dogs) compared to species that lose them early in life is of particular interest. Despite growing interest, the precise function of TIE2 + NP cells remains debated. Conflicting results, methodological inconsistencies, and species-specific variations continue to complicate interpretations of its function.

This scoping review consolidates the current knowledge on TIE2 + NP cells, analyzing their purpose through a better understanding of their presence (The Who), characteristics (The What), and functional significance across species (The Why). A central objective of this review is also to critically appraise the reproducibility of reported findings across species, laboratories, and methodologies, and to distinguish consistently replicated observations from those that remain preliminary or unconfirmed. While identifying key research gaps and methodological challenges, we aim to clarify the biological relevance of TIE2 in NP homeostasis and explore its potential as a therapeutic target. Uniquely, this review also compares the regulation of TIE2 in the NP with its canonical role in angiogenesis and tumor biology, fields where TIE2 is well-studied. By contrasting TIE2 signaling across vascular and avascular environments, we highlight both conserved and divergent mechanisms that may redefine our understanding of TIE2 as a context-dependent regulator in progenitor biology. Finally, we discuss translational challenges and future research directions to harness their regenerative potential in particular in the context of IDD and aging (Table 1).

**Table 1:** Overview of the terminology of progenitor and TIE2 + cells used in the manuscript

Term	Description
TIE2 + cells	Cells specifically sorted or selected based on their presentation of TIE2 as a cell surface receptor. Depending on the setup, this term also represents cells that were TIE2 + at the onset of the experiment or cell selection
TIE2-enhanced cell populations	A cell product cultured or processed in an optimized method known or validated to enhance the TIE2-positivity rate (compared to standard culture methods [21]). The resulting cells have not been sorted and thus are not exclusively TIE2+
A progenitor cell population	A specific cell population with a confirmed progenitor cell-like phenotype, including high proliferative capacity, colony-formation abilities, and multi-lineage differentiation potential (commonly adipo-, chondro-, osteo-, and/or neurogenic). For the NP, this involves NPPCs. Note that this population may or may not include TIE2 + cells. Though NPPCs are linked with TIE2 positivity, a TIE2 positive cells does not always function as a progenitor cell and vice versa

Abbreviations: *NP* Nucleus pulposus; *NPPC* Nucleus pulposus progenitor cells; *TIE2* Tyrosine kinase with immunoglobulin-like and EGF-like domains 2 receptor

### Nomenclature

The research on TIE2 in NP cells encompasses a variety of studies [8, 17, 18, 22–53] that explore the role of TIE2 in different contexts and for various purposes. Consequently, the terminology used can vary significantly and become confusing. In this review, TIE2 refers to the protein, while *TEK* denotes its gene. Thus, an increased *TEK* expression indicates higher mRNA levels, whereas increased TIE2 reflects an increase in the receptor protein. While NPPCs are linked to TIE2-expression, not all TIE2+ cells function as progenitor cells and vice versa. For a complete definition overview, see Table 1. Throughout this review, we adhere to these specific definitions to ensure clarity, and we encourage those working with TIE2+ NP cells or NPPCs to do the same in future studies.

## Discovery of TIE2 + progenitor cells in the nucleus pulposus

The original discovery of TIE2 as the marker of a specific NPPC population was made by Sakai et al. in 2012 [8]. Initially, using mouse-derived NP cells, the authors identified NPPCs through a methylcellulose-based colony-forming assay (CFA). The colony-forming units (CFUs) were highly enriched in the cell surface receptor disialoganglioside (Gd2). These Gd2-positive NP cells formed colonies with a predominantly spherical morphology (as opposed to a fibroblastic morphology) and possessed a cellular phenotype indicative of active NP cells. When the authors repeated their CFA using human NP cells, they similarly found that sorting cells on GD2 yielded a higher number of spherical CFUs, whereas sorting on TIE2 yielded even higher spherical colony-forming abilities. Human TIE2 + NP cells formed spherical colonies that stained strongly positive for type II collagen and aggrecan, suggesting ECM remodeling potential. The rate of TIE2 + NP cells isolated from human disc tissues was strongly and negatively correlated with donor age and degeneration severity, underscoring a decline in TIE2 + cell yields with increasing age and Pfirrmann grade [8, 54]. Noteworthy is that overall NP cell numbers decrease with age-related degeneration; thus, the decline in TIE2 + NP cells occurs both proportionally and in absolute terms. Following subcutaneous transplantation experiments in immunodeficient mice demonstrated that only TIE2 + NP cells, and not cluster of differentiation (CD) 24 + NP cells (standard marker for active NP cells [55–57]), engrafted in irradiated young human NP tissue scaffolds were able to maintain the NP tissue graft.

Additionally, they demonstrated high self-renewal capacity, capable of repopulating ectopic NP tissue grafts, thereby validating their progenitor cell characteristics [8]. Subsequent harvesting of the engrafted cells and repeating the experiment with the collected cells showed the seeded TIE2 + NPPCs could still repopulate and maintain a second-generation graft while retaining full multi-lineage differentiation potential. These findings underscored the crucial role and high potency of TIE2 + cells in NP tissue maintenance and regeneration, underlining their NPPC-like phenotype. However, no subsequent studies have independently replicated this specific transplantation and differentiation experiment. While these findings suggest a strong regenerative potential for TIE2 + NPPCs, further validation is needed to confirm their long-term functionality and therapeutic applicability. Since this discovery, multiple studies have recognized TIE2 as a potent marker of an NPPC population [19, 55, 58, 59]. In turn, research has aimed to harness or target this cell population to produce regenerative therapies possibly alleviating discogenic LBP and halting or reversing IDD, which are discussed later.

## Characteristics of TIE2 + NP cells

### *Proliferative capacity and colony-forming potential of TIE2* + *NP cells*

TIE2 + NP cells were initially characterized by their ability to form high rates of spherical CFUs. In CFA, TIE2 + NP cells typically exhibit a population with a higher proportion of spherical CFUs compared to fibroblastic-like CFUs. Regarding the proliferative potential of TIE2 + NP cells, studies have repeatedly shown a correlation between increased TIE2 expression and increased levels of stem cell-related transcription factors such as *OCT4*, *NANOG*, and *SOX2* [38, 42, 43]. Additionally, Wangler et al. [33] identified a link between TIE2 + NP cells and the anti-apoptotic B-cell CLL/Lymphoma-2 (BCL2) expression, suggesting that these cells may possess enhanced survival potential. Concurrently, multipotent stromal cell homing [4, 60], which enhanced the TIE2 + cell population, was associated with an overall increase in proliferating Ki-67-positive disc cells. Moreover, Bischof et al. [45] observed that in highly expanded NP cell cultures, only TIE2 + NP cells could significantly grow in their 7-day culture, while TIE2- or mixed NP cell populations did not.

**Fig. 2:**
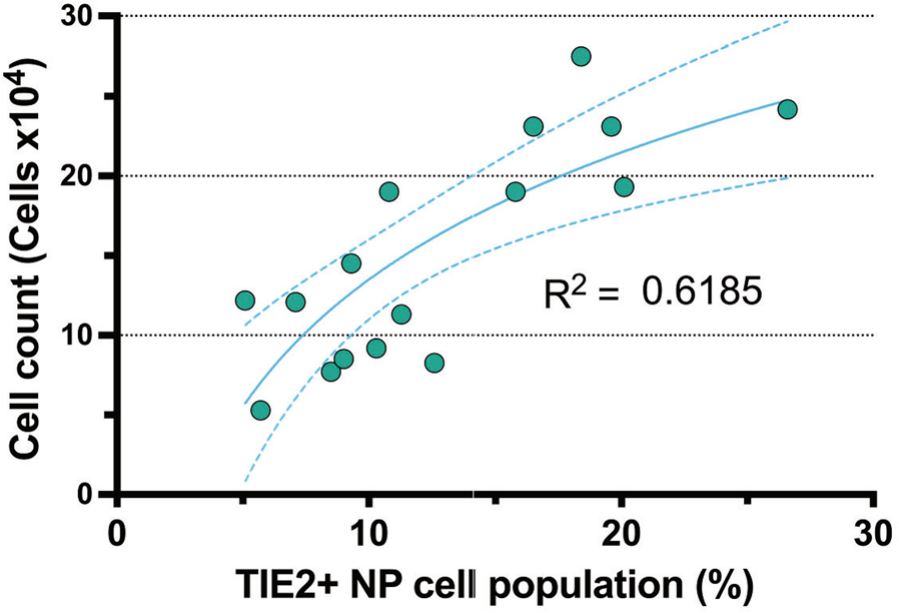
Correlation assessment of TIE2-positivity with cell count. The TIE2 positivity rate of an NP cell population correlated with the resulting NP cell yields as a measure of proliferation capacity, showing a moderate correlation with enhanced cell counts resulting from TIE2 enriched NP cell populations. Dots represent single measurements of a separate human donor. The line represents semi-log correlation trend with dashed lines representing 95% confidence intervals. This data is an original observation, provided to illustrate and support trends described in the literature. Abbreviations: *NP* Nucleus pulposus; *TIE2* Tyrosine kinase with immunoglobulin-like and EGF-like domains 2 receptor

For this review, we sought to confirm these trends and assessed the impact of TIE2 positivity on overall cell yields (Supplemental item 1). Using a limiting dilution approach (0.25 cells/well) to isolate progenitor-derived clones, we cultured human NP cells under optimized conditions and assessed TIE2-enhanced cell populations via flow cytometry (FCM). (Supplemental item 1) We found that clonal NP cell populations with enhanced TIE2 positivity presented with a higher number of overall NP cells yields (Fig. 2). Specifically, we found a moderate semi-log correlation with overall TIE2 positivity and overall NP cell numbers (Fig. 2). These findings further support the literature’s view that TIE2 + NP cells exhibit a progenitor-like phenotype, characterized by higher proliferative activity, thereby distinguishing them from the general NP cell population.

### *Differentiation potential of TIE2* + *NP cells*

Contemporary data leave the full differentiation potential of TIE2 + NP cells uncertain, as research has primarily examined human NP cells and their adipogenic, osteogenic, and chondrogenic potential. This trilineage potential is a common method for assessing MSCs [61], often referred to as mesenchymal stromal cells or mesenchymal stem cells, despite ongoing debates about the accuracy of these terms [62]. These multi-differentiation assays generally result in successful trilineage differentiation, with TIE2 + NP cells often outperforming TIE2- NP cells [17, 31, 32] and even show general higher chondrogenic and osteogenic differentiation potential compared to bone marrow-derived MSCs [38]. TIE2 + NP cells can undergo adipogenesis, but their capacity appears to be limited compared to MSCs [8, 32, 38]. Additionally, human NPPCs obtained after culture in TIE2-enhancing media have also shown potential for neurogenic differentiation. Ishii et al. [22] successfully promoted their NPPC population to adopt a neurogenic glial cell phenotype, resulting in a functional cell product capable of resolving sciatic nerve damage in their mouse model.

Finally, TIE2 + or TIE2-enhanced NP cells possess the obvious ability to form differentiated NP cells (e.g., CD24 + and beyond [8]) and maintain functionality and viability in the harsh NP environment [2, 3, 63, 64]. Importantly, it is critical to distinguish NP cells from general chondrocyte-like cells such as articular chondrocytes, as they arise from different embryonic origins, namely notochord versus paraxial mesoderm [19], and exhibit distinct phenotypes [18, 19, 65]. For instance, NP cells favor a higher glycosaminoglycan-to-collagen production ratio compared to articular chondrocytes [66]. Furthermore, their coping mechanisms in hypoxia differ markedly: NP cells constitutively express hypoxia-inducible factor 1α (HIF-1α), regardless of oxygen concentration in vitro, whereas articular chondrocytes modulate HIF-1α expression depending on oxygen levels [67–69]. Therefore, to fully validate the presence of TIE2 + NPPCs, authors could aim for quin-lineage differentiation: (i) adipogenic, (ii) osteogenic, (iii) neurogenic, (iv) chondrogenic, and (v) discogenic. However, discogenic differentiation remains poorly defined, as no clear consensus exists on assay outcomes distinguishing NP cells from general chondrogenic phenotypes, despite reported phenotypical differences [21, 55].

### *Cell surface marker profile of TIE2* + *NP cells*

TIE2 + NP cells and NPPCs have been characterized by various cell-surface markers. (Supplemental item 2) Most studies report that this subset of NP cells is positive for NP-associated markers [55] such as CD44, CD73, CD90, and CD105, and portions also show positivity for NP markers CD49f, CD56, CD166, and CDH2. Xia et al. [24] found that embryonic mouse-derived NP cells were negative for NP markers CD29, CD73, and CD105, suggesting that mouse notochordal cells might not align with standard human NP cell phenotypes. Nevertheless, Gao et al. [23] were able to detect CD44, CD73, CD90, and CD105 on NP cells from their young mice NP tissue. Rodrigues-Pinto et al. [35] observed that CD90 was absent in early embryonic notochordal tissues, with CD24 and CD55 appearing later in IVD development, indicating the importance of developmental timing in marker expression. Additionally, NP markers and receptors detected in NPPCs show high overlap with specified MSC markers (CD73, CD90, and CD105), and reports show low expression of negative MSC markers (CD14, CD34, and CD45), suggesting NPPCs may possess phenotypical characteristics resembling MSCs. Evidence supports that TIE2 + NP cells are likely specific to the NP tissue rather than contaminants from surrounding structures rich in TIE2-expressing blood vessels, such as the endplates or the annulus fibrosus (AF). For example, CD146, a potent AF marker [70], was negative in all studies that examined it. CD31 and the von Willebrand factor (VWF), endothelial cell markers, were also negative, as reported by Sakai et al. [8], with subsequent studies finding that NPPC populations were negative for CD34, alleviating concerns of sample contamination by vasculature tissue [8, 27, 34, 40, 41, 43]. However, the possibility of contamination from the endplate tissue remains because specific endplate-cell markers are lacking, prompting further investigation.

### TIE2 regulation and functional role in NP tissue maintenance

Studies have consistently shown a preference for basic fibroblastic growth factor (FGF-2) stimulation in maintaining TIE2 positivity [31, 46, 47], while other factors such as epidermal growth factor (EGF), vascular endothelial growth factor (VEGF), platelet-derived growth factor (PDGF), growth differentiation factor 5 (GDF5), growth differentiation factor 6 (GDF6), and transforming growth factor beta 1 (TGF-β1) do not achieve this effect [31, 46]. However, Frapin et al. [30] demonstrated that the combined administration of TGF-β1 and GDF5 augmented the TIE2 positivity rate within their ex vivo cultured ovine NP tissue. Notably, previous reports [71, 72] have highlighted a synergistic effect of TGF-β1 and GDF5, although it remains unclear how this affects TIE2 regulation. Environmental conditions also play a significant role. For instance, serum starvation notably reduced TIE2 positivity [46]. In contrast, increased medium osmolarity (including 10% fetal calf serum) during expansion led to a remarkable enhancement in TIE2 positivity in chondrodystrophic Beagle dog-derived NP cells, which was maintained during a re-differentiation culture in serum-free medium supplemented with TGF-β1 [36]. Hypoxic conditions show a context-dependent effects on TIE2 regulation. While some NP cell cultures retain TIE2 expression under hypoxia [31], others show little benefit [36], and have been linked with limited proliferation, though supporting *ACAN* and *COL2A1* expression in human TIE2 + NP cells [45]. Furthermore, evidence from HIF-1α manipulation studies supports a regulatory link i.e., enhancing HIF-1α activity can protect NPPCs from apoptosis [27]. Conversely, oxidative stress appears to have the opposite effect, reducing TIE2 positivity while leaving other NP markers relatively unchanged [50]. Collectively these findings highlight that hypoxia signaling redox conditions contribute directly to maintaining the TIE2 + NPPC pool, but their influence is highly dependent on species, culture conditions, and cell state.

Finally, TIE2 + NP cells exhibit a distinct molecular profile characterized by high expression of progenitor markers and are functionally associated with enhanced NP ECM production. Colonies derived from TIE2 + NP cells exhibit high positivity for aggrecan and type II collagen [42, 47, 51]. Specifically, NP cell populations showing enrichment in TIE2 positivity correlate strongly with the proportion of NP cells positive for intracellular type II collagen [42, 47, 51] and with increased expression of *ACAN* and *COL2A1* [36, 42]. Moreover, extracellular vesicles (EVs) [73] derived from human TIE2-enhanced NP cells were more effective in promoting cell viability and disc repair compared to those from non-TIE2-enhanced NP cells or MSC populations [74]. Furthermore, a recent study by Zhang et al. [28], successfully reprogrammed degenerative human NP cells into a notochordal-like phenotype through the overexpression of *OCT4*, *TBXT*, and *FOXA2*. This phenotype was validated using sophisticated techniques, including single cell RNA-sequencing (scRNA-seq) analysis, which identified TIE2 and *TEK* as key markers for their classified “homeostatic” notochordal cell cluster. This population, enriched in *TEK* expression, exhibited high levels of anabolic factors such as *COL2A1*, *SOX9*, and *ACAN*. Furthermore, overexpression *of Oct4*, *Tbxt*, and *Foxa2* in a rat IDD model promoted the formation of notochordal cell-like cells enriched in Tie2 expression, which, in turn, alleviated IDD, as demonstrated by improved radiographic and histological outcomes. More recent data indicate that TIE2 positivity may persist across a wider range of degeneration severities than previously appreciated, albeit sometimes with reduced staining intensity [53]. These contrasting observations underscore that the relationship between TIE2 expression, progenitor activity, and disc pathology is not yet fully resolved. Taken together, the available evidence supports TIE2 as a marker associated with anabolic activity, ECM production, and progenitor-like behavior in NP cells, but highlights the need for more rigorous and standardized approaches to clarify how TIE2 + cells contribute to NP homeostasis and whether they represent an optimal therapeutic target in IDD.

## Principles of TIE2 signaling from angiogenesis to NP homeostasis

### Lessons that can be learned from the regulation of TIE2 in angiogenesis

**Fig. 3:**
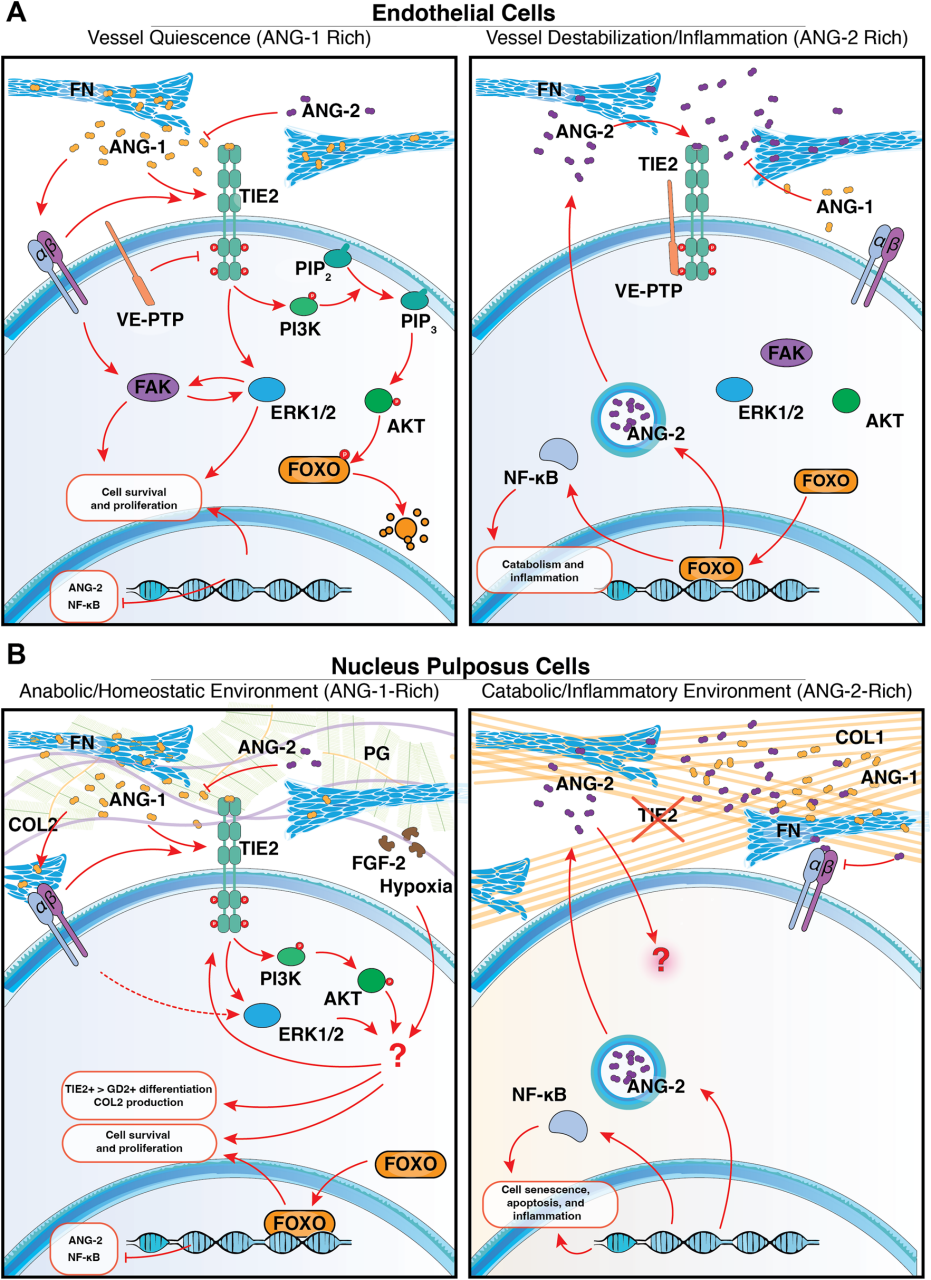
The TIE2 regulation comparison within discal and angiogenic niches. TIE2 regulation or signaling pathway in **A** endothelial cells and **B** nucleus pulposus cells. Abbreviations: *ANG-1* Angiopoietin-1; *ANG-2* Angiopoietin-2; *COL1* Type I collagen; *COL2* Type II collagen; *ECM* Extracellular matrix; *ERK1/2* Extracellular signal-regulated kinases 1 and 2; *FAK* Focal adhesion kinase; *FGF-2* Basic fibroblast growth factor; *FN* Fibronectin; *FOXO* Forkhead box O transcription factors; *GD2* Disialoganglioside GD2; *NF-κB* Nuclear factor kappa-light-chain-enhancer of activated B cells; *PG* Proteoglycan; *PI3K* Phosphoinositide 3-kinase; *PIP*_*2*_ Phosphatidylinositol 4,5-bisphosphate; *PIP*_*3*_ Phosphatidylinositol (3,4,5)-trisphosphate; *TIE2* Tyrosine kinase with immunoglobulin-like and EGF-like domains 2 receptor; *VE-PTP* Vascular endothelial-protein tyrosine phosphatase

TIE2 is integral to vasculogenesis, the embryonic development of vasculature, and angiogenesis, the remodeling of the vasculature to construct new vessels based on regional needs. TIE2 primarily modulates endothelial cell behavior through its interactions with angiopoietins, specifically angiopoietin-1 (ANG-1) and angiopoietin-2 (ANG-2) [6, 75], which are crucial for endothelial cell proliferation, migration, adhesion, and survival [5, 6, 75] (Fig. 3).

A key feature of vascular TIE2 regulation is its context-dependence. ANG-1 promotes endothelial quiescence by clustering TIE2 with regulators such as vascular endothelial-phosphotyrosine phosphatase (VE-PTP) at endothelial cell–cell junctions, whereas ECM-bound ANG-1 activates TIE2 on migrating endothelial cells through integrin and focal adhesion kinase (FAK) signaling [75–78]. This illustrates two core principles: TIE2 activity is strongly shaped by the local ECM composition and by the density of TIE2-expressing neighboring cells. Both principles are likely to be highly relevant for NP cells, where ligand availability, ECM interactions, and cell density change markedly with degeneration [8, 79–81]. ANG-2 adds a second layer of context specificity. In most blood vessels ANG-2 antagonizes ANG-1 and suppresses TIE2 activation, yet in lymphatic endothelium, where VE-PTP is absent, ANG-2 functions as an agonist and promotes vessel formation [13, 82]. This ligand switching demonstrates that TIE2 output is not hardwired, but depends on the surrounding molecular environment [83, 84]. As such, the ANG-1/ANG-2 signaling axis is a significant therapeutic target for vascular pathologies. Particularly, the inhibition of ANG-2 has emerged as a promising strategy in the development of clinical interventions [76, 85, 86]. For example, the usage of monoclonal antibodies targeting ANG-2 e.g., faricimab, has shown promising results in clinical trials for treating retinal vascular diseases [87].

In addition to VE-PTP, TIE2 also interacts with other key receptors such as tyrosine kinase with immunoglobulin-like and EGF-like domains 1 (TIE1), vascular endothelial growth factor receptor 2 (VEGFR2), and integrins, which collectively modulate angiogenesis, vascular remodeling, and endothelial cell adhesion [75–78]. Such variability is important when considering the NP, where neither VE-PTP nor classical endothelial junctional complexes have been identified.

Downstream, ANG-1-driven activation of phosphatidylinositol 3-kinase (PI3K)/protein-kinase B (AKT) and the extracellular signal-regulated kinases (ERK)-1/2 stabilizes endothelial cells. Particularly, ANG-1-mediated activation of TIE2 involves a cascade of p85, p110 and AKT activation, which in turn (amongst other factors) phosphorylate the transcription factor FOXO-1, − 3, and − 4 [12], preventing them from activating *ANGPT2* (gene of ANG-2) amongst other targets [88] (Fig. 3). Moreover, receptor activation similarly upregulates the mitogen-activated protein kinases (MAPK) pathway, promoting proliferation and preventing nuclear factor kappa-light-chain-enhancer of activated B cells (NF-κB) activation, averting an inflammatory phenotype [12, 88] (Fig. 3). These signaling axes overlap substantially and integrate inputs from multiple receptors and environmental cues. Organ-specific angiogenesis studies therefore demonstrate that dysregulation of TIE2 can lead to divergent biological outcomes even within the same organism [89, 90]. This reinforces the expectation that the NP, with its unique hypoxic, high-pressure, and avascular niche, may employ distinct variants of TIE2 regulation rather than strictly mirroring endothelial biology. Indeed, notochordal cell-derived factor studies have shown that these factors are able to modulate angiogenic pathways in both inhibitory and stimulatory directions [91–93]. This highlights the likelihood that ANG–TIE2 signaling behaves differently in the disc and vascular tissues.

Collectively, angiogenesis research demonstrates that TIE2 signaling is shaped by ligand balance, ECM interactions, and local cellular context, resulting in markedly different biological outputs across tissues. Importantly, these studies show that divergent outcomes arise not from distinct signaling machinery, but from differential use of shared, highly interconnected pathways. This conceptual framework is directly relevant to the NP, where hypoxia, matrix composition, and cell density differ fundamentally from vascular environments. Rather than predicting an endothelial-like behavior, angiogenesis literature therefore provides mechanistic principles that help explain why TIE2 regulation in the NP must be experimentally interrogated within disc-specific conditions.

### Overlapping mechanisms of TIE2 in angiogenesis and NP homeostasis

**Fig. 4:**
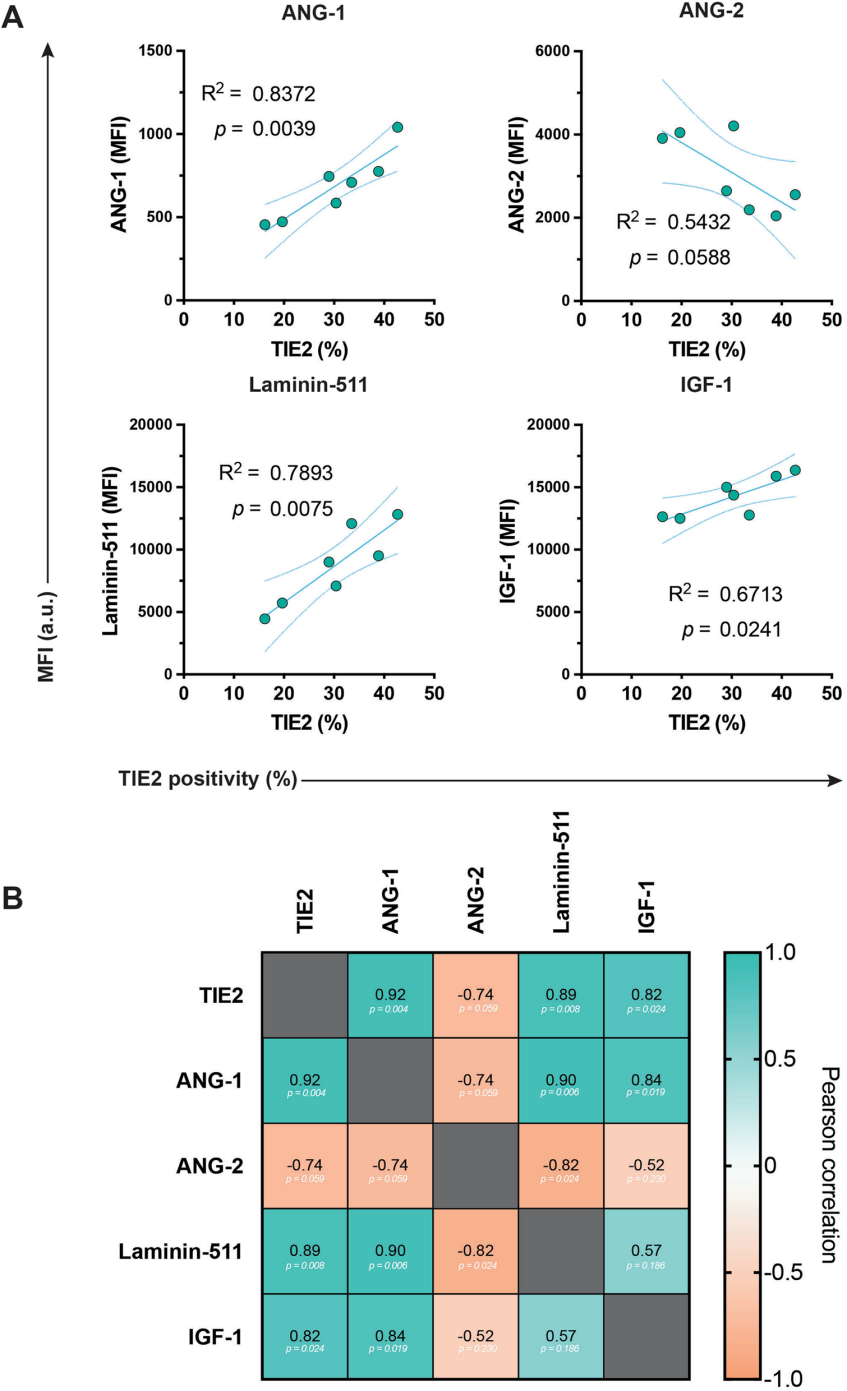
Correlation assessment of TIE2 positivity with intracellular protein production. **A** Linear correlation assessment between the TIE2-positivity rate of NP cell clones and the respective MFI of known signaling proteins in the TIE2-related pathways, as assessed through flow cytometry. Dot represents single measurements of a separate NP cell clone. Lines represent correlation trend with dashed lines representing 95% confidence intervals. **B** Pearson correlation matrix of TIE2 positivity, ANG-1 MFI, ANG-2 MFI, Laminin-511 MFI, and IGF-1 MFI. Data included are original observations which are provided to illustrate and support trends described in the literature. Abbreviations: *ANG-1* Angiopoietin 1; *ANG-2* Angiopoietin 2; *a.u.* arbitrary unit; *IGF-1* Insulin growth factor 1; *MFI* Mean fluorescence intensity; *NP* Nucleus pulposus; *TIE2* Tyrosine kinase with immunoglobulin-like and EGF-like domains 2 receptor

**Fig. 5:**
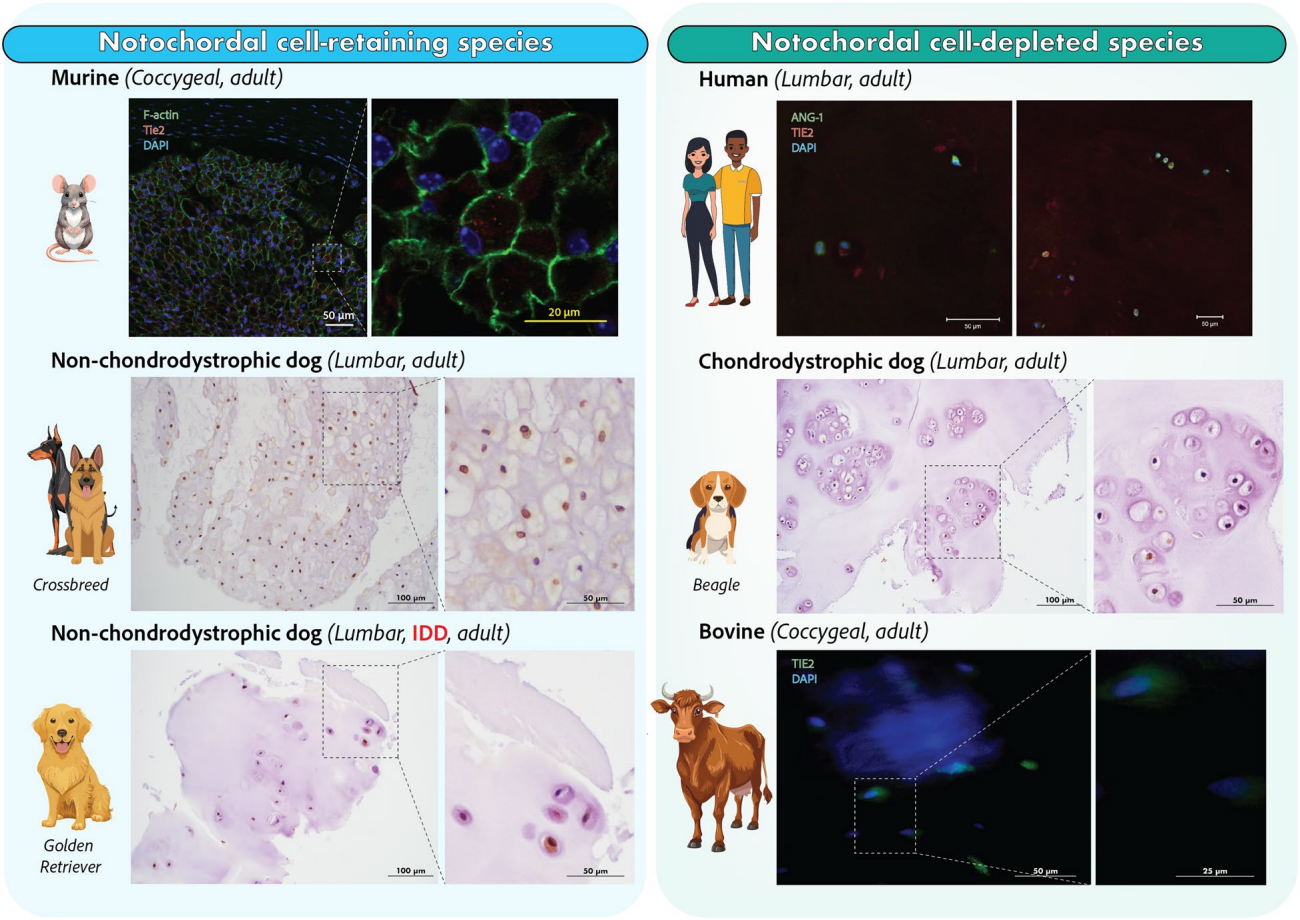
Overview of stained tissues of variety of notochordal-retaining or notochordal-depleted IVD tissues. Selection of successfully stained TIE2 proteins in the IVD specimens across different species. Note, the bottom left panel under “notochordal cell-retaining species” shows tissue from a client-owned Golden Retriever (10 years old) presenting with severe IDD and LBP in predilection sites (cervical and lower lumbar regions, similar to human IDD). Although non-chondrodystrophic by breed, this dog had lost the predominance of notochordal cells in the NP tissue as part of the degenerative process. For isotype controls, please refer to Supplemental item 8. Abbreviations: *Ang-1* Angiopoietin 1; *DAPI* 4’,6-diamidino-2-phenylindole; *IDD* Intervertebral disc degeneration; *IVD* Intervertebral disc; *LBP* Low back pain; *TIE2* Tyrosine kinase with immunoglobulin-like and EGF-like domains 2 receptor

The regulation of TIE2 in NP cells remains incompletely defined, but several lines of evidence indicate partial conservation with vascular biology alongside clear features. In the human NP tissue, ANG-1 colocalizes with TIE2 + cells and increases as TIE2 + /GD2 − cells progress through the GD2-positive differentiation hierarchy [8] (Fig. 5). This suggests that ANG-1 may participate in a positive feedback loop that supports NPPC activation and early differentiation [8] (Fig. 1). Consistently, ANG-1 supplementation enhances spheroid colony formation, and TIE2 blockade prevents this effect [8]. Furthermore, previously unpublished data show that our optimized culture method [47] followed by FCM [17], likewise demonstrates a strong correlation between TIE2 positivity and intracellular ANG-1 levels (Fig. 4, see supplementary item 1 for methods).

In contrast, ANG-2 is consistently associated with degenerative and catabolic NP cell states [39, 94, 95]. While ANG-1 and *ANGPT1* expression remained relatively stable with IDD [39], studies demonstrate increased ANGPT2 expression in human IDD tissue and in NP cells exposed to mechanical or inflammatory stress. Moreover, ANG-2 directly promotes apoptosis, matrix breakdown (by increasing matrix metalloproteinase-13 [MMP13] and a disintegrin and metalloproteinase with thrombospondin motifs-4 [ADAMTS4] expression), and interleukin (IL)-1β production [39, 94, 95]. Our dataset similarly identifies a negative correlation between ANG-2, anabolic markers, and TIE2. Similarly, our previously unpublished FCM data suggest a moderate-to-strong negative correlation between ANG-2 production and anabolic protein expression, such as ANG-1, laminin-511, and insulin growth factor-1 (IGF-1), as well as TIE2 (Fig. 4B). A key uncertainty remains through which receptors ANG-2 acts in the NP, since TIE2 + cells decline sharply with age and degeneration [8, 17]. Although some tissues exhibit ANG-2 driven suppression of *TEK* expression, others do not [96], and our observations support an inverse relationship between ANG-2 levels and TIE2 + NP cell abundance. Whether ANG-2 directly limits TIE2 + cell maintenance or whether TIE2-enriched populations restrain ANG-2 expression remains unresolved.

Based on current evidence, reduced ANG-1 availability in the NP niche could contribute to early loss of TIE2 + cells and acceleration of NPPC maturation. ANG-1 and TIE2 + NP cells colocalize [8], yet this association has not been functionally tested. Another possibility is that NP cells employ a regulation scheme more similar to the lymphatic endothelium, where ANG-2 acts independently of VE-PTP [82]. The strong negative association between ANG-2 and TIE2 (Fig. 4B) in our data argues against ANG-2 acting as a TIE2 agonist in the NP but highlights the need to determine whether additional ligands, phosphatases, or co-receptors modulate TIE2 under disc-specific conditions.

Downstream signaling studies further support partial overlap with endothelial mechanisms, though the signaling cascade underlying TIE2 activity within the NP has only been examined in a handful of studies (Fig. 3). Within NP cells, interference with PI3K/AKT and MAPK/ERK signaling reduces the proportion of human TIE2 + NP cells, without affecting GD2 or CD24 expressing NP cells, indicating these pathways are central to maintaining NPPCs [47]. Consistent with this, FGF-2 and engineered FGF-2 variants, augment ERK and AKT activation and support TIE2-enhanced human NP cell populations [31, 47]. Similarly, IGF-1, an activator of the PI3K/AKT and ERK pathways, has repeatedly been linked to anabolic NP activity [97–99], NP cell differentiation [99, 100], and stem cell maintenance [101]; although, contradictory reports are also present [102]. Our dataset likewise shows a strong correlation between TIE2 positivity and IGF-1 (Fig. 4), although the mechanistic relationship remains undefined. Together, these findings suggest that FGF-2 and IGF-1, both known activators of PI3K/AKT and ERK signaling, may support pathways that favor anabolic activity and help sustain progenitor-like features. While direct evidence linking these factors to TIE2 + NPPC maintenance remains limited, their shared downstream signaling provides a plausible mechanism worth further investigation.

FOXO transcription factors, although not directly examined in TIE2 + NPPCs, are well-established downstream targets of AKT signaling and play key roles in NP homeostasis. In mice, *FOXO-1* and *FOXO-3* loss leads to early NP cell apoptosis and consequential IVD deterioration [103]. Moreover, FOXO activity contributes to oxidative stress resistance, autophagy, and adaptation to hypoxia [103, 104] (Fig. 3). This diverges from endothelial biology, where FOXO primarily upregulates ANG-2 during catabolic stress. One possible explanation for this divergence is the unique hypoxic environment of the NP, where constitutive HIF1α activity [67, 68, 105] may alter FOXO transcriptional output and suppress ANG-2–associated responses and represents an important direction for future research.

ECM-dependent signaling also appears central to TIE2 regulation. ANG-1 is able to bind to fibronectin (FN) within the NP ECM and can promote the formation of TIE2–integrin (α5β1) complexes, activating FAK and PI3K/AKT signaling to support NP cell survival and proliferation [39]. In contrast, ANG-2 competes with ANG-1 for FN binding, disrupting FAK and PI3K/AKT signaling, thereby potentially inducing a catabolic NF-κB–driven phenotype [39, 94]. Laminin-rich environment can further reinforce NPPC features e.g., TIE2 + NP cells cultured on laminin-511 show enhanced NPPC behavior combined with upregulated α3 and α6 integrin and ERK activation [48]. The work of Bischof et al. [45] presents a contrasting perspective, where both ANG-1 and ANG-2 were applied to expanded human TIE2+ , TIE2-, and unsorted NP cell populations, resulting in minimal effects on NP cells regardless of TIE2 expression. However, the lack of ECM coating and the use of expanded, dedifferentiated NP cells likely contributed to these discrepancies, as TIE2 signaling appears highly context-dependent and may require appropriate ECM ligands or progenitor cell states to be biologically active [51].

Taken together, the available evidence suggests that TIE2 activity in NP cells involves regulatory interactions that differ from those described in endothelial systems, reflecting both shared and tissue-specific aspects of TIE2 function. NPPC maintenance appears to rely on PI3K/AKT and ERK signaling and is highly dependent on ECM interactions, particularly with FN and laminins. ANG-1 consistently promotes NPPC survival through these pathways, whereas ANG-2 can destabilize them under certain conditions. Conflicting findings across studies likely arise from differences in ECM composition, culture conditions, and the differentiation state of NP cells. Although mechanistic details remain incomplete, a working model emerges in which TIE2 integrates growth-factor signaling and ECM cues to maintain a progenitor-like NP phenotype.

## TIE2 + NP cells across experimental systems and development

### *TIE2* + *NP cells *in vitro

TIE2 + NP cells have been investigated in vitro to clarify their characteristics, regulation, and potential therapeutic applications. Across multiple species, TIE2 + or *TEK*-expressing NP cells have been identified using antibody-based and RNA-based techniques, although several studies failed to detect these markers under certain conditions (Supplemental item 3). These inconsistencies underscore the need for a deeper understanding of the factors influencing TIE2/*TEK* expression in vitro. Because both the number [8] and potency [51] of TIE2 + NP cells decline with age and degeneration, their scarcity presents a challenge for translational applications and highlight the importance of optimized expansion strategies. The following section reviews in vitro findings, with attention to regulatory patterns and sources of methodological variation.

**Fig. 6:**
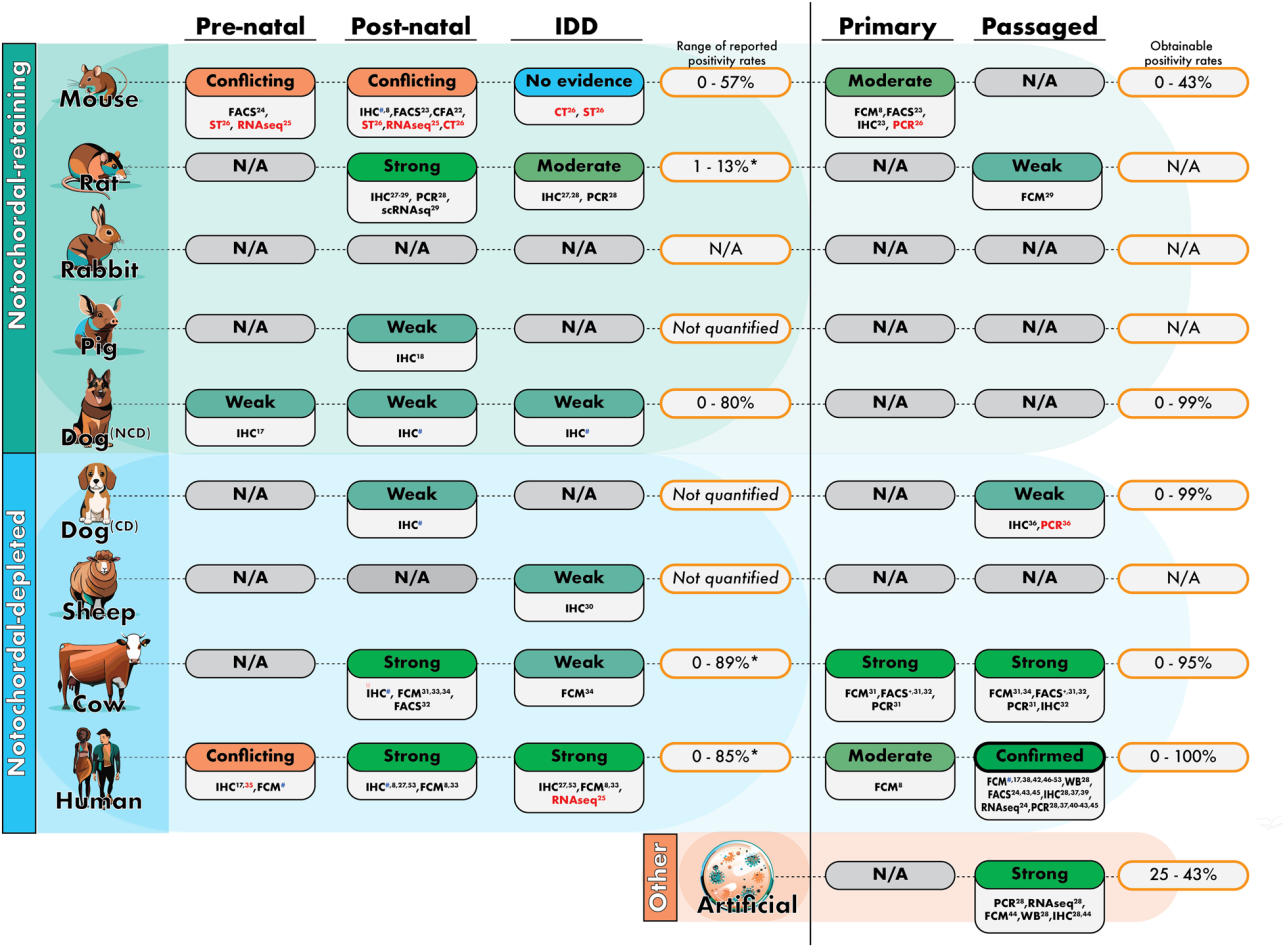
Overview of the level of evidence and studies confirming TIE2 + NP cells. Detection (or lack thereof) is presented for tissue detection or as observed during in vitro cell cultures, summarizing Supplemental item 3 and 5. Images includes original observational data provided to illustrate and support trends described in the literature. Reference indicated in “red” involve studies that were unable to detect TIE2 or TEK. (^+^) Assessment of FACS, MACS, and PluriSelect as a functional sorting method, (*) Positivity highly dependent on donor age and IDD stage, (^#^) Supportive evidence provided in this review. Abbreviations: *CD* Chondrodystrophic; *CFA* Colony-forming assay; *CT* Cell tracing; *FACS* Fluorescence-activated cell sorting; *FCM* Flow cytometry; *IDD* Intervertebral disc degeneration; *IHC* Immunohistochemistry; *N/A* Not applicable; *NCD* Non-chondrodystrophic; *NP* Nucleus pulposus; *PCR* Polymerase chain reaction; *RNA-seq* RNA sequencing; *ST* Spatial transcriptomics; *TIE2* Tyrosine kinase with immunoglobulin-like and EGF-like domains 2 receptor; *WB* Western blot

Across species, most studies report detectable TIE2 or *TEK* expression in cultures, including mouse [8, 23], rat [29], dog [36], bovine [31, 32, 34], and human samples [24, 37, 38, 40–43, 45–53] (Fig. 6). However, two studies were unable to detect *TEK* transcripts in their NP cells (Supplemental item 2). Laagland et al. [36] observed near-complete TIE2 positivity in Beagle dog NP cultures under high-osmolarity conditions, yet *TEK* mRNA remained undetectable. Primer validation confirmed the primers should recognize dog *TEK.* (Supplemental item 1 and 4) Although the primers potentially could detect *SCN9A*, *ZZNF62*9, and *ZN275,* cross-reactivity is unlikely since these genes are not recognized by both forward and reverse primers (Supplemental item 4). Chen et al. [26] similarly reported undetectable *Tek* expression in murine NP cultures, even after FGF-2 treatment. However, BLAST analysis of their primer sequences for *Tek* (Supplemental item 4) failed to produce hits for *TEK*, suggesting that the reported absence of detectable *Tek* may reflect a methodological limitation. This could relate either to assay sensitivity or to an unintended error in the primer sequence described in the Materials and Methods. Nonetheless, the discrepancy between TIE2 positivity and *TEK* expression, as reported by Laagland et al. [36], has also been seen in other studies (Supplemental item 3). Zhang et al. [42] found significant increases in TIE2 positivity using spheroid-based culture methods, yet *TEK* expression did not show clear changes. These patterns indicate that *TEK* transcript levels often do not correlate directly with TIE2 protein expression. Possible explanations include low *TEK* transcript abundance, rapid receptor trafficking independent of new transcription, or technical limitations of NP RNA detection [106]. Collectively, these findings suggest that *TEK* mRNA is a less reliable marker for defining TIE2 + NPPCs, whereas the TIE2 protein remains a more dependable readout.

A consistent observation across species is the rapid decline of TIE2+ NP cells during conventional 2D culture [53]. In mouse NP cells, Tie2 expression drops from ~ 8% at day 10 to near zero by day 30, [8] with similar declines reported in bovine [107] and human cultures [37, 51]. Age further influences retention: Otani et al. [51] reported greater loss of TIE2 positivity in cells from older donors. Furthermore, several groups have shown that specific culture conditions, including FGF-2 supplementation [31, 46, 47], increased osmolarity [36], hypoxia [31, 46, 47], and 3D matrices such as spheroids [42, 46], whole-tissue culture [17, 47, 51], or alginate beads [37, 43], enhance the proportion and stability of TIE2 + NP cells. This sensitivity to environmental cues reinforces the concept that TIE2 expression is strongly microenvironment-dependent and may require ECM support, mechanical context, and local growth factor niche to remain stable. However, the optimization of culture conditions is out of the scope for this work but merits an in-depth analysis in a separate review.

Cell reprogramming studies offer further insights while introducing additional complexity. Differentiation of human embryonic pluripotent stem cells (ESC) and human induced pluripotent stem cells (iPSC) toward a notochordal-like phenotype produced heterogeneous cell populations with 25–43% TIE2 positivity [44], although *TEK* transcript levels did not clearly distinguish TIE2 + from TIE2- subsets. In the context of in vitro differentiation of pluripotent stem cells, TIE2 expression needs to be interpreted with care. The differentiation of pluripotent stem cells generally creates a mixed cell population of different lineages and TIE2 is also expressed by a plethora of other cell types like glial cells and adipocytes [108]. In line with this, Zhang et al. observed that *TEK* expression did not differ prior to and after differentiation between the TIE2/GD2-positive cells compared to TIE2/GD2-negative cells [42]. Furthermore, the field lacks a specific marker that can uniquely and exclusively identify NP cells in the stages of development, from vacuolated embryonic to mature notochordal cells to non-vacuolated NP cells still capable of producing the appropriate ECM, which hampers the exact determination of the differentiation efficiency. A separate study overexpressing OCT4, TBXT, and FOXA2 in degenerative NP cells (both human and rat) induced notochordal-like characteristics, accompanied by increased *TEK* and TIE2 expression [28]. Together, these findings support the concept that TIE2 + NP cells represent a transitional population that may emerge during shifts between notochordal, progenitor-like, and mature NP states. However, definitive lineage relationships remain unresolved and will require dedicated single-cell and lineage-tracing approaches.

Together, the in vitro evidence indicates that discrepancies between *TEK* transcript detection and TIE2 protein expression most likely arise from technical sensitivity, low *TEK* mRNA abundance, and context-dependent receptor regulation. Across studies, TIE2 protein emerges as the most reliable indicator of progenitor-like NP cells. The rapid loss of TIE2 + cells ex vivo, unless supported by niche-like biochemical or biomechanical cues, further suggests that TIE2 marks a microenvironment-dependent, transitional NPPC state rather than a stable, fixed cellular identity.

### Expression of TIE2 in pre-natal and post-natal NP tissue

Since the original discovery of TIE2 + NP cells within the IVD of both mice and humans by Sakai et al. [8], numerous studies have examined their presence across species, including mouse [8, 23, 24], rat [27–29], pig [18], dog [17], sheep [30], cow [31–34], and human [8, 17, 27, 28, 33, 41, 53] tissues (Supplemental item 5). Representative immunohistochemistry (IHC) staining from our groups (previously unpublished; Fig. 5) illustrates the presence of TIE2 + cells in NP tissue of various species.

Most studies using antibody-based detection (e.g., IHC, fluorescence activated cell sorting [FACS], and FCM) report clear TIE2 + cells in rat, sheep, pig, dog, and bovine NP tissues. A multinational guideline paper on notochordal cell isolation [18] demonstrated strong TIE2 positivity in the porcine notochordal cell–rich NP, while Frapin et al. [30] reported similar findings in sheep and noted age-related declines in TIE2 + cells. They further showed that treatment of degenerative ovine discs with adipose-derived MSCs or CCL5/GDF5/TGF-β1 increased TIE2 positivity, suggesting that both notochordal-retaining and notochordal-depleted species can maintain substantial TIE2 + NP cell populations, although exact quantification was not reported [18, 30].

Bovine NP tissue is widely used to study TIE2 + cells, yet reported positivity rates vary markedly across studies [31–34]. Some groups identified only about 10% of TIE2 + cells in young bovines [31, 32], whereas others found values approaching 90% in similarly aged animals, with steep declines in older donors [34]. Additional work using long-term explant cultures reported even lower rates (2–4%) [33]. These discrepancies likely reflect methodological differences: for example, studies employed distinct tissue digestion protocols (e.g., type II [31, 32] and type XI [34] collagenases), used different antibody clones for TIE2 detection, and varied in whether tissues were analyzed immediately or after extended explant culture. Breed-related variation between Swiss [31, 32] and Portuguese [34] cattles may further contribute to those discrepancies [109]. Taken together, these factors suggest that differences in processing, detection sensitivity, and sampling conditions, rather than true biological variation, may account for the wide range of reported TIE2 + frequencies in bovine NP tissues.

Among smaller animal models and notochordal cell-retaining species, results are also mixed. In rats, Tie2 + NP cells were detected in lumbar and coccygeal discs with declines associated with aging and induced degeneration (to roughly 1%) [27]. Conversely, Tie2 levels were shown to increase through experimentally reactivating a notochordal-like phenotype, which was linked with improved disc repair [28]. Xue et al. [29] reported high Tie2/Gd2 co-expression in rat NP cells described by the authors as stem cells and identified a *Tek*-high cluster by scRNA-seq, specifically 1.5% of the NP cells were found to express high levels of *Tek* and *B4galnt1* (the gene encoding the enzyme involved in Gd2 biosynthesis). In mice, antibody-based methods have repeatedly detected Tie2 + NP cells [8, 23], yet transcriptomic studies including scRNA-seq and spatial transcriptomics have not detected *Tek* expression [25, 26].

Chen et al. [26] further reported the absence of *Tek*-lineage cells in the NP using a *Tek*-Cre reporter mouse, concluding that prior reports of Tie2 presence in murine NP cells/tissues were likely due to potential contamination from Tie2 + non-NP cell types [26]. This is difficult to reconcile with multiple reports of Tie2 + cells in the murine NP based on IHC, Tie2-GFP reporter mice [24], and studies showing that Tie2 + NP cells produce aggrecan and type II collagen. Promoter activity in Cre-based lineage tracing may be context-dependent [24, 110], and *Tek* mRNA abundance appears low in NP cells, potentially falling below detection thresholds of transcriptomic methods. Developmental timing and tissue handling may also contribute. Together, these factors suggest that methodological sensitivity, rather than true biological absence, may explain the inconsistent detection of *Tek* in the mouse NP.

Because TIE2 has been proposed to mark progenitor-like or transitional NP cell states, its developmental expression pattern is of particular interest; however, studies of pre-natal and early post-natal tissues have yielded conflicting results across species. Tan et al. [25] and Chen et al. [26] did not detect *Tek* expression in murine embryos or neonatal NP samples, whereas Xia et al. [24] observed strong Tie2 expression in the embryonic notochord and reported approximately 57% of cell to be Tie2-positive through FACS analysis. In dogs, fetal NP tissue from several breeds (Including Golden Retriever, Labrador, Crossbreed [mix of Labrador Golden and Hannoverscher Schweisshund]) samples; all non-chondrodystrophic dogs) displayed high TIE2 positivity [17]. Human data are similarly contradictory: Sakai et al. [17] detected TIE2-positive cells in roughly half of fetal discs, whereas Rodrigues-Pinto et al. [35] reported none, although the latter did not present their TIE2-stained images. Differences in antibodies, tissue processing, and transient developmental expression may explain these discrepancies.

Despite inconsistent reports, antibody-based studies indicate that TIE2-positive cells can be detected in both prenatal and postnatal human NP tissue, including cells with notochordal-like and fibrocartilage-like morphology. Reported detection rates vary substantially, likely reflecting small sample sizes, differences in tissue quality and processing, and technical challenges, particularly for RNA isolation from the NP tissue [111, 112] (Supplemental items 6 and 7). Observations from embryonic tissues, fetal dog NP, and iPSC-derived notochordal-like cells [44], consistently indicate that TIE2 expression increases during transitions between notochordal and NP phenotypes, supporting the idea that TIE2 marks a progenitor-like or transitional state. Across species and developmental stages, antibody-based methods reliably detect TIE2 + cells. Collectively, these findings suggest that TIE2 identifies a context-dependent progenitor-like NP cell population whose detectability is strongly influenced by developmental stage, cell state, and the methods used for tissue processing and molecular analysis.

## Limitations and considerations

In line with our objective to critically appraise the reproducibility of reported findings, this review highlights that evidence for TIE2 expression in the NP is strongest when supported by antibody-based detection across multiple species and laboratories, whereas *TEK* transcript detection remains inconsistent and often irreproducible due to methodological constraints. Findings that have been replicated across independent groups, species, and platforms consistently support the presence of a TIE2-positive NP subset, while reports that rely on single methodologies or unvalidated assays show greater variability and remain provisional. By integrating data from diverse models, processing protocols, and detection approaches, the collective evidence suggests that TIE2 + NP cells are present but difficult to be detected reliably, and that much of the apparent contradiction in the field reflects technical sensitivity rather than fundamental biological disagreement. Moreover, we have included some previously unpublished observations in this review intended to illustrate concepts and support trends identified across the literature, specifically in Figs. 2, 4, and 6. These should be interpreted only as complementary examples rather than primary evidence, and they do not serve as the basis for any major conclusions drawn in the manuscript.

One significant limitation is the heterogeneity in the methods used across studies. The techniques employed to detect TIE2 + or *TEK*-expressing cells varied widely, including IHC, quantitative polymerase chain reaction (qPCR), FCM, and others. This variation was compounded by differences in the antibodies used, concentrations applied, tissue or cell processing protocols, and primer sequences, all of which complicate direct comparisons between studies. For example, Sakai et al. [17] demonstrated that antibody specificity for the TIE2 receptor varies across species. Antibody selection is therefore critical, as differences in epitope recognition and limited cross-species validation can significantly affect detection reliability. Rigorous validation remains necessary to ensure accurate interpretation. Additionally, the presentation of outcomes was inconsistent, with some studies reporting fold increases while others used relative expression metrics, making it challenging to draw clear conclusions. Furthermore, many studies lacked comprehensive control groups or did not account for potential confounding factors, such as the influence of cell culture conditions [31, 42, 113], age-related variability in cell populations [38, 51], or the effects of long-term storage on cell viability and marker expression [46, 49, 114].

Finally, the variability in animal models used across studies introduces additional complexity. Differences in species, age, breeds, methods of cell harvest and culture may influence the outcomes, and caution must be taken when extrapolating these findings to human conditions. The same is true when translating findings from humans to animal models. Future research should focus on addressing these limitations by standardizing methods [18, 21] and employing rigorous controls (e.g., including images of isotype stainings as negative controls [see supplemental item 8] and endothelial rich-tissues as TIE2-positive controls [53])). Furthermore, the role of TIE2 and TIE2 + cells application in vivo should be carefully assessed to better understand the potential of TIE2 + NP cells in disc homeostasis, as therapeutic targets, and as a regenerative cell or cell-derived therapy [74, 115]. Another key limitation in TIE2 detection arises from the lack of isoform-specific information provided by antibody manufacturers, as TIE2 exists both as a full-length membrane-bound receptor (~ 150 kDa) and as a soluble isoform (e.g., 75 kDa) [116]; this ambiguity necessitates more conscientious consideration in experimental design and data interpretation.

## Applications, future directions, and conclusion

Despite notable progress, there is still room for discovery and deeper understanding of TIE2 + NP cells, particularly regarding the regulation and function as NPPCs in vivo. Future research should focus on elucidating the mechanisms that control their behavior and regulation within their native environment, which will be essential for both supporting their therapeutic applications and optimizing in vitro culture conditions preserving TIE2 expression and their regenerative potential [19].

Although some studies were unable to show TIE2 or *TEK* expression, the majority of reported studies demonstrate the presence of TIE2 + NP cells in the NP tissue. This seemingly contradiction should be interpreted cautiously. While evidence in dogs, pigs, and sheep is less comprehensive, the consistent presence of TIE2 + NP cells in more extensively studied species, i.e., mice, rats, cows, and humans, suggests a similar role across these animals. A better understanding of the role of TIE2 + NPPCs in the transition from vacuolated notochordal cells to non-vacuolated NP cells could provide critical insights, particularly through comparative studies across species that either maintain or lose vacuolated notochordal cells with aging. It is tempting to speculate that TIE2 + cells may represent a transitional progenitor population that emerges in response to changes in the disc environment, bridging the decline of vacuolated notochordal cells and the establishment of the adult NP cell phenotype. Speculatively, anabolic factors, shifts in ECM composition, and niche-specific ECM interactions, as well as adaptations to hypoxia, may critically influence the behavior and fate of these TIE2 + cells during this transition. Given the limited current understanding, this represents an exciting field for further investigation.

In addition to TIE2, a variety of other markers have recently been reported, highlighting new and additional markers to classify stem/progenitor cells of the NP [58]. Specifically, recent transcriptomic data from various studies have suggested *Uts2r* (in mice) [23], *CTSK* (in mice and human) [26, 117], and *TAGLN* (in human) [25] as potential markers of an NPPC phenotype. It is interesting that these studies report mixed results with regard to TIE2 as an additional receptor to identify the progenitor population. For example, the work of Gao et al. [23] who discovered Uts2r as a marker for their mice-derived NPPCs, showed a very high (approximately 64%) Tie2 positivity in the *Uts2r* + NPPCs. Contradictory, Chen et al. [26] suggested *Ctsk* as a NPPC marker and reported only rare presence of *Utsr2* and no expression of *Tek*. *CTSK* was also identified by Zhou et al. [117] in human and mice embryos but these authors did not mention TIE2 or UTS2R in their report, or equivalent gene expression. How these different markers represent distinct or overlapping cellular populations, or how they might be hierarchically linked, requires further studies.

IDD-associated LBP remains notoriously difficult to treat and is significantly on the rise due to increasing life expectancy and obesity rates [118, 119]. With current clinical strategies limited to conservative pain management or, at more severe stages, surgical spinal fusion [120, 121], there is an urgent need for novel regenerative approaches [122]. Targeting disc-specific cell populations represents a promising strategy to restore the catabolic environment of the degenerating disc. In particular, the TIE2 + NPPC population holds strong potential, as these cells could provide a sustained source of metabolically active NP cells to support disc repair. Direct transplantation of TIE2 + NPPCs, or their induction *in-situ* through gene therapy-based reprogramming, may help replenish and expand the pool of progenitor cells, thereby restoring the functional NP cell population lost as part of if IDD [28, 51, 52]. Alternatively, TIE2 + cells may serve as a valuable source for tissue engineering applications [123], or for harvesting cell-derived products such as EVs, ECM, and other biologics [73, 124]. Another promising avenue is the *in-situ* activation or modulation of endogenous TIE2 + cells using gene therapy [125, 126], biomaterial-based approaches [126–128], and other biologics [128]. However, such strategies are likely to be most effective in younger individuals or in the early stages of IDD, when these cells are still present and retain at least partial activity, as their abundance and function become markedly compromised with age and disease progression [8, 51]. Which of these strategies will ultimately prove most effective remains to be determined through future research. At present, the reintroduction of TIE2 + NP cells as a cell-based therapeutic represents the most advanced strategy considering their development status, with encouraging results reported in large animal models and preparations underway for clinical translation [51, 115].

A better understanding of TIE2 + NP cells regulation, function, and their location in the differentiation and maturation hierarchy will be a benefit to enhance their utilization in this aspect. The regulation of TIE2 in the NP warrants further investigation, particularly as its signaling mechanisms appear to diverge from those predominantly characterized in vascular biology. This suggests a tissue-specific regulatory network that remains largely unexplored. Additionally, while ANG-1 and ANG-2 have been the primary focus due to their established roles in vascular and cancer research, less studied ligands such as ANG-3 and ANG-4 may potentially play a significant role in NP-specific TIE2 signaling and warrant further studies. Also, creating a niche environment for TIE2 + NP cells to maintain their progenitor state, for both optimizing in vitro culture or as a cell-based therapy, by for example optimizing biomaterial carriers [129–132] or through growth factor stimulation [72, 130], might further help enhance their potency as a regenerative product.

## Supplementary Information

Additional file 1.

Additional file 2.

Additional file 3.

Additional file 4.

Additional file 5.

Additional file 6.

Additional file 7.

Additional file 8.

## Data Availability

The data presented in this review are fully available in the manuscript and accompanying supplemental files.
