# Supplementary material for: TIE2-positive cells in the nucleus pulposus with a purpose: the who, what and why
Source: J Biomed Sci. 2026 Mar 2;33:24. doi: 10.1186/s12929-026-01220-7 (PMC12952123; doi:10.1186/s12929-026-01220-7)
Supplement: Supplementary file 1 — Additional file 1. [file 12929_2026_1220_MOESM1_ESM.pdf]

Supplemental data

Supplemental item 1. Overview of Methodological Processes Involved in the Review.

8. Methods and Guidelines

For this review we aimed to adhere to the suggestions of the ORS Spine sections, in reference to the nomenclature and minimal reporting criteria suggested in the consensus paper of Basatvat et al. [1] regarding the NP cells and Williams et al. [2] with regard to notochordal cells.

8.1 Reverse primer BLAST

Primer sequences used to assess *TEK* expression were evaluated for their specificity to *TEK* mRNA variants. Using the BLAST® system provided by the U.S. National Library of Medicine (https://blast.ncbi.nlm.nih.gov/blast/Blast.cgi; accessed 2025/10/14), the primer sequences were input, and corresponding nucleotide sequences were retrieved for the organism specified in the associated paper. Max scores and E-values for the primary hits were recorded. Only mRNA (NM) or predicted mRNA (XM) hits were considered, excluding sequences from Bacterial Artificial Chromosomes (BACs), P1-derived Artificial Chromosomes (PACs), or "AL" sequences. Additionally, only BLAST® hits with an E-value below 10 were reviewed, unless they were related to *TEK*.

8.2 Immunohistochemistry

The images of IHC staining included in figure 6 are derived from previous work. For full methods, see the work of Sakai et al. [3] (mice and human) or Laagland et al. [4] (dog). In short, human samples involve lumbar specimen of disc herniation stained with antihuman TIE2/TEK mAb (Upstate Millipore, Ab33), anti-human angiopoietin-1 rabbit anti-sera (Acris Antibodies, GmbH), and DAPI. Mouse sample involves a 9-week-old coccygeal specimen in an axial plane stained with anti-mouse TIE2/TEK biotinylated goat pAb (R&D Systems), Alexa Fluor-488 conjugated phalloidin (targets F-actin fibers; Invitrogen), and DAPI.

Bovine IVDs were isolated and fixed in 4% formaldehyde (VWR International, limited, Dietikon, Switzerland, Cat. 9713.9010) for 24 h. Samples were washed three times in phosphate-buffered saline (PBS) and were subjected to sequential incubation in 15% and 30% (w/v) sucrose in PBS at 4 °C until saturation. Samples were then embedded in optimal cutting temperature (OCT) compound (Tissue-Tek®, Alphen aan den Rijn, The Netherlands) and frozen in liquid nitrogen. 12 µm-sections were blocked with PBS containing 10% fetal calf serum (FCS). Sections were incubated overnight at 4 °C with a rabbit polyclonal anti-TIE2/CD202b antibody (Bioss, distributed by Bio-Connect, inc., Oss, The Netherlands, bs-1300R; 1:200 dilution in PBS containing 0.5% bovine serum albumin [BSA], Sigma-Aldrich, Buchs, Switzerland). Samples were washed with PBS and incubated for 2–3 h at room temperature with Alexa Fluor 488-conjugated goat anti-rabbit secondary antibody (Molecular Probes, A-11008; 1:200 dilution in PBS with 0.5% BSA). Nuclei were counterstained using SlowFade™ Gold Antifade Mounting Media containing DAPI (Molecular Probes, Thermo-Fisher Scientific, Basel, Switzerland, Cat. S36939), and coverslips were mounted. Slides were examined using a fluorescent microscope within 7 days.

8.3 Cell culture

To assess the correlation of TIE2 positivity with (i) cell yields and (ii) signaling protein expression, we cultured human NP cells following previous work. [5] These tissues were collected as part of

standard surgical procedures against lumbar disc herniation and were approved by the Tokai University School of Medicine Institutional Review Board for Clinical Research (approval number 17R-173). Informed written consent was obtained from every involved patient. Cells were isolated and cultured in accordance with the work of Sako et al. [5] to obtain a NP cell population with enhanced TIE2 positivity. In brief, in accordance with the recommendation set forth in the work of Basatvat et al. [1], the collected NP tissue was segmented, washed, and directly cultured in TUNZ Pharma optimized media blend, comprising 20% fetal bovine serum (FBS; Gibco, USA), 32% Minimum Essential Medium α (MEMα; Gibco), and 48% (v/v) Dulbecco's Modified Eagle Medium low glucose (DMEM; WAKO, Japan) mixed with 1% penicillin/streptomycin. These whole tissue cultures (WTC) were incubated for 2 weeks without media refresh under 5% CO_2 and 5% O_2.

Following WTC [5], the cells were extracted through sequential digestion with TrypLE Express (ThermoFisher, USA) for 1 hour and 0.25 mg mL^{-1} Collagenase P for 2 hours. The resulting cell suspension was filtered using a 40 µm cell strainer and cultured in TUNZ Pharma optimized media blend [5] supplemented with 10 ng mL^{-1} FGF-2 (PeproTech, USA) for an additional week on poly-L-Lysine-coated plasticware (IWAKI, Japan) at a density of about 550 cells cm^{-2}. The cultures were kept at 5% CO_2 and 5% O_2. The final products were detached and collected using TrypLE Express for 5 minutes.

The resulting cell suspension were then counted, and a limiting dilution (at 0.25 cell/well) method was used to obtain progenitor cell derived clones. The seeded clones were cultured in previously defined culture conditions (TUNZ Pharma optimized media blend) and resulting clonal cell cultures were examined through FCM to determine which (intracellular) factors (i.e., ANG-1, ANG-2, Laminin-511, and IGF-1) were associated with enhanced TIE2 positivity of the clonal products.

8.4 Flow cytometry analysis

The collected cells from two donors (18- and 19-year-old males) were carefully washed and processed for FCM analysis in line with previous recommendations. [6,7] Cell surface marker TIE2 was detected using mouse anti-human-TIE2 antibody conjugated with APC-labels (monoclonal IgG1, FAB3131A; R&D Systems, USA). [6] For intracellular staining of ANG-1, ANG-2, Laminin-511, and IGF-1, the antibodies mouse monoclonal anti-human Angiopietin-1 (R&D Systems; Cat. No. MAB923), mouse monoclonal anti-human Angiopietin-2 (R&D Systems; Cat. No. MAB098), mouse monoclonal anti-human laminin-511 (Funakoshi, Japan; Cat. No. FDV-0026), and rabbit polyclonal anti-human IGF-1 pAb (Abcam, United Kingdom; Cat. No. AB9572) were used respectively. As a secondary either Alexa488 goat anti-mouse IgG (Invitrogen, USA; Cat. No. A28175) or Alexa488 goat anti-rabbit IgG (Invitrogen; Cat. No. A11008) was applied. For intracellular staining, the cells were fixed and permeabilized using the IntraPrep Permeabilization Reagent kit (A07803; Beckman Coulter, USA).[7] FCM analysis was performed using a FACS Calibur flow cytometer (BD Biosciences, USA), applying propidium iodide staining to exclude non-viable cells.

8.5 Data collection, statistical analysis, writing, and illustrations

Data from reviewed papers was collected in excel and processed as desired. Reports presenting data of interest in graphs only, were retrieved by using WebPlotDigitizer version 4.4 (https://automeris.io by A. Rohatgi) to estimate reported averages and standard deviations.

Supplemental data

Correlation analysis was performed using GraphPad Prism version 10.3.1 (GraphPad Software LLC). Results presented in figure 4A involved a simple linear regression analysis. Results presented in figure 4B involved the assessment of correlation by defining Pearson correlation coefficients. Gaussian distribution was assumed. For figure 2 involved a semi-log correlation. For our work a p-value below 0.05 was considered statistically significant. A correlation coefficient of $R^2 > 0.4$ was classified as moderate, while $R^2 > 0.7$ was regarded as strong. Figure 1 was produced using the BioRender platform (http://www.biorender.com). All remaining figures were drawn or composed using Adobe Illustrator version 27.8.1. (Adobe Inc., USA). Illustrations within the figures were generated using AI-assisted tools available within the Adobe software suite.

Supplemental data

REFERENCES

1 Basatvat, S. *et al.* Harmonization and standardization of nucleus pulposus cell extraction and culture methods. *JOR Spine* **6**, e1238, doi:10.1002/jsp2.1238 (2023).

2 Williams, R. J. *et al.* Recommendations for intervertebral disc notochordal cell investigation: From isolation to characterization. *JOR Spine* **6**, e1272, doi:10.1002/jsp2.1272 (2023).

3 Sakai, D. *et al.* Exhaustion of nucleus pulposus progenitor cells with ageing and degeneration of the intervertebral disc. *Nat Commun* **3**, 1264, doi:10.1038/ncomms2226 (2012).

4 Laagland, L. T. *et al.* Hyperosmolar expansion medium improves nucleus pulposus cell phenotype. *JOR Spine* **5**, e1219, doi:10.1002/jsp2.1219 (2022).

5 Sako, K. *et al.* Effect of Whole Tissue Culture and Basic Fibroblast Growth Factor on Maintenance of Tie2 Molecule Expression in Human Nucleus Pulposus Cells. *International journal of molecular sciences* **22**, doi:10.3390/ijms22094723 (2021).

6 Sakai, D. *et al.* Successful fishing for nucleus pulposus progenitor cells of the intervertebral disc across species. *JOR Spine* **1**, e1018, doi:10.1002/jsp2.1018 (2018).

7 Soma, H. *et al.* Recombinant Laminin-511 Fragment (iMatrix-511) Coating Supports Maintenance of Human Nucleus Pulposus Progenitor Cells In Vitro. *International journal of molecular sciences* **24**, doi:10.3390/ijms242316713 (2023).
