# Supplementary material for: TIE2-positive cells in the nucleus pulposus with a purpose: the who, what and why
Source: J Biomed Sci. 2026 Mar 2;33:24. doi: 10.1186/s12929-026-01220-7 (PMC12952123; doi:10.1186/s12929-026-01220-7)
Supplement: Supplementary file 2 — Additional file 2. [file 12929_2026_1220_MOESM2_ESM.pdf]

Supplemental data

Supplemental item 2. Overview of the Different Cell Surface Receptors Assessed in Relationship with Nucleus Pulposus (NP) Progenitor Cell Populations or TIE2+ NP Cells.

Author	Year	Ref	Species (Condition)	STRO-1	CD146	CD90	CD73	CD105	CD44	CD56	CDH2	GD2	CD24	CD166	CD49f	USTR	CD49e	CD55	Lectin	CD11b/c	CD144	VWF	CD31	CD29	CD34	CD14	CD19	CD45	HLA-DR
Xia	2023	1	Mouse (TD)				-	-	+		+		+											-					
Gao	2022	2	Mouse			+	+	+	+							+													
He	2021	3	Rat			+	+		+																-				-
Xue	2024	4	Rat									+	-																
Laagland	2022	5	Dog			+	+						+																
Molinos	2023	6	Bovine	-					+			-												+	-				
Zeng	2020	7	Human			+	+	+	+				-							-				+	-	-		-	-
Soma	2023	8	Human									-	-		+		+												
Wu	2018	9	Human			+	+	+	+				-							-				+	-	-		-	-
Guerrero	2020	10	Human		-	+	+	+																	-			+	
Sakai	2012	11	Human		-	+	+	+	+	+		+	-	+	+				-	-	-	-	-		-	-		-	
MSC markers		12	Human			+	+	+																	-	-	-	-	-
NPC markers		13	Human			+	+	+	+	+	+	+	+	+	+														
EC markers		14	Human		+		+	+													+	+	+	+	+				
SC markers		15	Human			+							+	+	+								+	+					

NP cell population determined (largely) ■ positive or ■ negative for indicated receptor/marker. Determined ■ positive or ■ negative marker for the indicated population following the referenced guidelines or reviews. ■ indicates items that were not mentioned or not found evidently positive/negative. Abbreviations: EC – Endothelial cell, MSC – Multipotent stromal cell, NP – Nucleus pulposus, SC – Stem cell, and TD – Tissue-derived

REFERENCES

1 Xia, K. S. *et al.* An esterase-responsive ibuprofen nano-micelle pre-modified embryo derived nucleus pulposus progenitor cells promote the regeneration of intervertebral disc degeneration. *Bioact Mater* **21**, 69-85, doi:10.1016/j.bioactmat.2022.07.024 (2023).

2 Gao, B. *et al.* Discovery and Application of Postnatal Nucleus Pulposus Progenitors Essential for Intervertebral Disc Homeostasis and Degeneration. *Adv Sci (Weinh)* **9**, e2104888, doi:10.1002/advs.202104888 (2022).

3 He, R. *et al.* HIF1A Alleviates compression-induced apoptosis of nucleus pulposus derived stem cells via upregulating autophagy. *Autophagy* **17**, 3338-3360, doi:10.1080/15548627.2021.1872227 (2021).

4 Xue, B. *et al.* A Novel Superparamagnetic-Responsive Hydrogel Facilitates Disc Regeneration by Orchestrating Cell Recruitment, Proliferation, and Differentiation within Hostile Inflammatory Niche. *Adv Sci (Weinh)* **11**, e2408093, doi:10.1002/advs.202408093 (2024).

5 Laagland, L. T. *et al.* Hyperosmolar expansion medium improves nucleus pulposus cell phenotype. *JOR Spine* **5**, e1219, doi:10.1002/jsp2.1219 (2022).

6 Molinos, M. *et al.* Alterations of bovine nucleus pulposus cells with aging. *Aging Cell* **22**, e13873, doi:10.1111/acel.13873 (2023).

7 Zeng, X. *et al.* Effect of Conditioned Medium from Human Umbilical Cord-Derived Mesenchymal Stromal Cells on Rejuvenation of Nucleus Pulposus Derived Stem/Progenitor Cells from Degenerated Intervertebral Disc. *Int J Stem Cells* **13**, 257-267, doi:10.15283/ijsc20027 (2020).

8 Soma, H. *et al.* Recombinant Laminin-511 Fragment (iMatrix-511) Coating Supports Maintenance of Human Nucleus Pulposus Progenitor Cells In Vitro. *International journal of molecular sciences* **24**, doi:10.3390/ijms242316713 (2023).

9 Wu, H. *et al.* Regenerative potential of human nucleus pulposus resident stem/progenitor cells declines with ageing and intervertebral disc degeneration. *Int J Mol Med* **42**, 2193-2202, doi:10.3892/ijmm.2018.3766 (2018).

10 Guerrero, J., Hackel, S., Croft, A. S., Albers, C. E. & Gantenbein, B. The effects of 3D culture on the expansion and maintenance of nucleus pulposus progenitor cell multipotency. *JOR Spine* **4**, e1131, doi:10.1002/jsp2.1131 (2021).

11 Sakai, D. *et al.* Exhaustion of nucleus pulposus progenitor cells with ageing and degeneration of the intervertebral disc. *Nat Commun* **3**, 1264, doi:10.1038/ncomms2226 (2012).

12 Dominici, M. *et al.* Minimal criteria for defining multipotent mesenchymal stromal cells. The International Society for Cellular Therapy position statement. *Cytotherapy* **8**, 315-317, doi:10.1080/14653240600855905 (2006).

13 Risbud, M. V. *et al.* Defining the phenotype of young healthy nucleus pulposus cells: recommendations of the Spine Research Interest Group at the 2014 annual ORS meeting. *J Orthop Res* **33**, 283-293, doi:10.1002/jor.22789 (2015).

14 Goncharov, N. V. *et al.* Markers of Endothelial Cells in Normal and Pathological Conditions. *Biochem (Mosc) Suppl Ser A Membr Cell Biol* **14**, 167-183, doi:10.1134/S1990747819030140 (2020).

15 Zhao, W., Ji, X., Zhang, F., Li, L. & Ma, L. Embryonic stem cell markers. *Molecules* **17**, 6196-6236, doi:10.3390/molecules17066196 (2012).
