# Supplementary material for: TIE2-positive cells in the nucleus pulposus with a purpose: the who, what and why
Source: J Biomed Sci. 2026 Mar 2;33:24. doi: 10.1186/s12929-026-01220-7 (PMC12952123; doi:10.1186/s12929-026-01220-7)
Supplement: Supplementary file 3 — Additional file 3. [file 12929_2026_1220_MOESM3_ESM.pdf]

Supplemental data

Supplemental item 3. Overview of Reports Assessing the Presence of TIE2+ or *TEK*-Expressing Cells in vitro.

Species	Authors	Year	Ref	Life-stage	Culture	Method	Presen ce	Condition *	Positive rate †	Notes
**Mouse**	Sakai et al.	2012	[1]	Juvenile	Primary	FCM	✓	10-day culture	8.0% ± 2.7%	Positivity rates are Tie2/Gd2 double positivity rates
								14-day culture	14.5% ± 5.6%	
								30-day culture	~0%	
	Gao et al.	2022	[2]	Juvenile	Primary	FACS	✓	7-day culture	*See notes*	12% of Uts2r+ Pro-NPs-derived cells were Tie2+, 31% were Tie2+ Gd2+, and 54% were Tie2−, Gd2+
						IHC	✓	7-day culture	Not quantified	Evident Tie2+ cells, co-localized with Gd2. The rate of Gd2 went up and Tie2 went down with extended culture (From 0 to 7 days)
	Chen et al.	2024	[3]	Unspecified	Primary	PCR	✗	-	N/A	No *Tek* expression detected, but BLAST assessments suggest primers are not specific for *Tek* (See table 4)
**Rat**	Xue et al.	2024	[4]	Juvenile	Passaged	FCM	✓	Colony forming assay	99.1%	NPSCs did not express the NP marker Cd24, but showed high expression of Tie2 and Gd2
**Canine**	Laagland et al.	2022	[5]	Adult	Passaged	IHC	✓	300 mOsm/L	~0%	
								400 mOsm/L	~1%	
								500 mOsm/L	~100%	
						PCR	✗	-	N/A	No *TEK* was detected, but *ANGPT1* expression was high in the 500 mOsm/L culture media
**Bovine**	Tekari et al.	2016	[6]	Juvenile	Primary/passaged	FACS/FCM	✓	Primary cells 3 days culture	18.5% ± 4.3%	
								Primary cells 7 days culture	0.6% ± 0.3%	
								Primary cells in normoxia	0.8% ± 0.1%	
								Primary cells in hypoxia	3.3% ± 0.8%	
								Expanded cells in normoxia	1.05% ± 0.4%	
								Expanded cells with FGF-2	8.9% ± 2.6%	
								Expanded cells in hypoxia	8.4% ± 1.3%	
								Expanded cells with FGF-2 in hypoxia	17.1% ± 3.4%	
						PCR	✓	-	N/A	FGF-2, Hypoxia, or both increased *TEK* expression
	Frauchiger et al.	2018	[7]	Juvenile	Primary /Passaged	FACS, MACS, pluriSelect	✓	-	Not quantified	
						IHC	✓	Chondrogenic pellet cultures	Not quantified	TIE2+ pellets gave rise to pellets with much higher proteoglycan and aggrecan positive
	Molinos et al.	2023	[8]	Juvenile	Passaged	FCM	✓	Passage 1	~38%	
								Passage 2	~95%	
								Passage 3	~95%	
								Passage 4	~55%	
				Adult	Passaged	FCM	✓	Passage 1	~0%	Old bovine donors (10-16 year)
								Passage 2	~0%	
								Passage 3	~1%	
								Passage 4	~0%	
**Human**	Sakai et al.	2012	[1]	Juvenile to adult	Primary	FCM	✓	14-day culture	14.5% ± 5.6%	Positivity rates are TIE2/GD2 double positivity rates

	Sun et al.	2015	9	Adolescent	Monolayer to alginate beads	IHC	✓	7-day culture	~45%	
							✓	28-day culture	~25%	
						PCR	✓	-	N/A	*TEK* expression shows trend of increase with passaging (up to P3), *TEK* expression goes down in alginate beads
				Adult	Alginate beads	IHC	✓	7-day culture	~30%	
							✓	28-day culture	~25%	
						PCR	✓	-	N/A	*TEK* expression shows trend of decrease with passaging (up to P3), *TEK* expression goes up in alginate beads
	Li et al.	2017	10	Adult	Passaged	FCM	✓	-	14.1% ± 8.3%	
	Wang et al.	2017	11	Juvenile to adult	Passaged	IHC	✓	-	Not quantified	
	Sakai et al.	2018	12	Fetal	Passaged	FCM	✓	-	0% – 17%	
	Wu et al.	2018	13	Juvenile to adult	Passaged	PCR	✓	Low MSC-marker expression NPCs	N/A	
								High MSC-marker expression NPCs	N/A	*TEK* expression higher in NPC samples presenting high MSC markers (i.e., CD20 and CD105)
	Zeng et al.	2020	14	Adult	Passaged	PCR	✓	dNPCs	N/A	
								dNPCs + UC-MSC CM	N/A	*TEK* expression increased ~87-fold by UC-MSC conditioned media supplementation
	Zhang et al.	2020	15	Adult (nondegenerative)	Passaged	FCM	✓	Standard culture	10% ± 5%	
								Gelatin-coat culture	4% ± 5%	
								Spheroid culture	36% ± 16%	
								1st generation spheroids	30% ± 9%	
								2nd generation spheroids	43% ± 6%	
						PCR	✓	-	N/A	No clear difference in *TEK* expression between culture conditions, despite evident positivity changes
	Guerrero et al.	2020	16	Adult	Passaged	FACS	✓	Monolayer	~27% ± 5%	
								Alginate beads	45% ± 5%	
								Monolayer to FN-coating	~8% ± 17%	
								Alginate beads to FN-coating	75% ± 20%	
						PCR	✓	-	N/A	TEK expression in alginate beads showed 193-fold increase compared to monolayer. *TEK* in FN-coat was 2.5-fold higher if pre-cultured in alginate beads Osteogenic/adipogenic differentiation did not significantly alter *TEK* levels, but chondrogenic differentiation significantly increased *TEK*. TIE2+ and TIE2- sorted and cultured cells, in both monolayer and alginate beads significantly *TEK* levels for Tie2+ NP cells.
	Zhang et al.	2020	17	Not applicable	iPSC derived	FCM	✓		25% – 30%	iPSC-derived notochordal-like cells differentiated by TGF-β1 to a NP cell phenotype
						IHC	✓		43%	
	Bischof et al.	2021	18	Juvenile	Primary/Passaged	FACS	✓	-	Not quantified	

						PCR	✓	Variety of conditions *(2% or 21% O_2, with or without Ang-1 or Ang-2)*	N/A	*TEK* expression did not change, despite changes in oxygen tension, ANG-1 or ANG-2 supplementation at 10-100 ng mL^{-1} concentrations
	Sako et al.	2021	19	Juvenile	Passaged	FCM	✓	Variety of cryopreservation solutions (CB1, SCB, or SCBD-Free)	12.6%	A trend toward reduced TIE2- expression was found with CB1 compared with SCBD-Free, although this difference was not significant
	Sako et al.	2021	20	Adult	Passaged	FCM	✓	Standard	1.9% ± 0.3%	
								WTC	7.0% ± 2.6%	
								Standard + FGF-2	5.7% ± 2.0%	
								WTC + FGF-2	14.2% ± 5.4%	
								WTC to Monolayer	10.0% ± 3.2%	
								WTC to Monolayer + FGF-2	10.7% ± 2.1%	
								WTC to Monolayer + cFGF	16.3% ± 1.1%	
	Soma et al.	2023	21	Juvenile	Passaged	FCM	✓	Standard	7.1% ± 3.5%	
								Laminin-coating	19.4% ± 6.8%	
	Munesada et al.	2023	22	Adult	Passaged	FCM	✓	EDTA recovery	3.5% – 5.0%	
								HA-recovery	4.5% – 6.5%	
	Xia et al.	2023	23	Embryo	Passaged	RNA-seq	✓	3D culture in a U-bottom low attachment 96-well plate or normal 96-well plated pre-coated with Matrigel with DMEM/F12 supplemented with 0.5 µg/ml ascorbic acid–2-phosphate, 100 ng/mL FGF-2, 10 µM Y27632, 0.2% Heparin sodium, 10 nM CHIR99021, and 1 µM SB202190	N/A	*TEK* highly expressed in hNPPCs, which was reduced in passage 1 and increased in passage10
						FACS	✓		99.8%	
	Tamagawa et al.	2024	24	Adult	Passaged	FCM	✓	0mM Paraquat	17.2% ± 5.7%	Paraquat is used to induce oxidative stress, which impacts TIE2+ rates, but not GD2 or CD24-rates
								10mM Paraquat	4.6% ± 1.4%	
								30mM Paraquat	2.6% ± 1.6%	
	Otani et al.	2024	25	Adult	Passaged	FCM	✓	Young adult, 4 weeks:	20.8% ± 3.2%	
								Young adult, 5 weeks:	14.2% ± 2.1%	
								Old, 4 weeks:	17.5% ± 3.7%	
								Old, 5 weeks:	4.1% ± 2.9%	
	Zhang et al.	2024	26	Juvenile to adult	Passaged	IHC	✓	Normal NPCs	N/A	Low positivity
								Degenerative NPCs	N/A	Low positivity, which decreased further under 14 days 3D-differentiation conditions
				N/A	Reprogramming	IHC	✓	Induced notochordal cells	N/A	Low positivity which strongly increased under 14 days 3D-differentiation conditions, and followed strong increase in Aggrecan, KRT18, and type II collagen positivity
				Juvenile to adult	Passaged	WB	✓	Normal NPCs	N/A	Low but detectable TIE2, which was higher than degenerative NPCs

							✓	Degenerative NPCs	N/A	Low but detectable TIE2, which could be increased by applying differentiation conditions
				N/A	Reprogramming	WB	✓	Degenerative NPCs	N/A	Degenerative NPCs overexpressing *OCT4*, *TBXT*, and *FOXA2* showed strong TIE2 density, which was further enhanced in differentiation cultures.
				Juvenile to adult	Passaged	PCR	✓	Normal NPCs	N/A	Low but detectable *TEK*, which was higher than degenerative NPCs
							✓	Degenerative NPCs	N/A	Low but detectable *TEK*, which was not significantly increased by applying differentiation conditions
				N/A	Reprogramming	PCR	✓	Degenerative NPCs	N/A	Degenerative NPCs overexpressing *OCT4*, *TBXT*, and *FOXA2* showed enhanced *TEK* expression, but was strongly enhanced in differentiation cultures. *TEK* increase associated with *ACAN*, *COL2A1*, *SOX9*, and *KRT19* expression
						scRNA-seq	✓	Induced notochordal cells	N/A	TEK was identified as marker for induced notochordal cells, and moreover classified a specific cluster of homeostasis NP cells; which strongly expressed *SOX9* and *COL2A1*. Moreover, *TEK* expression was low for degenerative cells and its fibroblast and inflammatory clusters.
							✓	Degenerative NPCs to induced notochordal cells	N/A	The promotion of a notochordal phenotype from degenerative NP cells resulted in delayed increase in *TEK* which was lost at the end of the differentiation process. The *TEK* expression followed *COL2A1* levels.
	Ogasawara et al.	2024	[27]	Juvenile	Passaged	FCM	✓	-	20.1%	-
	Ionescu et al	2024	[28]	Juvenile to adult	Primary	FCM	✓	-	3.5% (0.5-13.9%)	-

* Description of culture conditions, if different conditions were employed in which Tie2 or TEK was assessed. † Values are presented as mean ± standard deviation or range values as indicated. A `~` symbol was used for estimated values taken from graphs included in the article. Abbreviations: FACS – Fluorescence activated cell sorting, FCM – Flow cytometry, IHC – Immunohistochemistry, N/A – not applicable (as PCR is unable to determine positivity rates), UC-MSC – Umbilical cord mesenchymal stromal cells.

REFERENCES

1 Sakai, D. *et al.* Exhaustion of nucleus pulposus progenitor cells with ageing and degeneration of the intervertebral disc. *Nat Commun* **3**, 1264, doi:10.1038/ncomms2226 (2012).

2 Gao, B. *et al.* Discovery and Application of Postnatal Nucleus Pulposus Progenitors Essential for Intervertebral Disc Homeostasis and Degeneration. *Adv Sci (Weinh)* **9**, e2104888, doi:10.1002/advs.202104888 (2022).

3 Chen, Y. *et al.* Characterization of the Nucleus Pulposus Progenitor Cells via Spatial Transcriptomics. *Adv Sci (Weinh)* **11**, e2303752, doi:10.1002/advs.202303752 (2024).

4 Xue, B. *et al.* A Novel Superparamagnetic-Responsive Hydrogel Facilitates Disc Regeneration by Orchestrating Cell Recruitment, Proliferation, and Differentiation within Hostile Inflammatory Niche. *Adv Sci (Weinh)* **11**, e2408093, doi:10.1002/advs.202408093 (2024).

5 Laagland, L. T. *et al.* Hyperosmolar expansion medium improves nucleus pulposus cell phenotype. *JOR Spine* **5**, e1219, doi:10.1002/jsp2.1219 (2022).

6 Tekari, A., Chan, S. C. W., Sakai, D., Grad, S. & Gantenbein, B. Angiopoietin-1 receptor Tie2 distinguishes multipotent differentiation capability in bovine coccygeal nucleus pulposus cells. *Stem cell research & therapy* **7**, 75, doi:10.1186/s13287-016-0337-9 (2016).

7 Frauchiger, D. A. *et al.* Fluorescence-Activated Cell Sorting Is More Potent to Fish Intervertebral Disk Progenitor Cells Than Magnetic and Beads-Based Methods. *Tissue Eng Part C Methods* **25**, 571-580, doi:10.1089/ten.TEC.2018.0375 (2019).

8 Molinos, M. *et al.* Alterations of bovine nucleus pulposus cells with aging. *Aging Cell* **22**, e13873, doi:10.1111/acel.13873 (2023).

9 Sun, Y. *et al.* Enrichment of committed human nucleus pulposus cells expressing chondroitin sulfate proteoglycans under alginate encapsulation. *Osteoarthritis and cartilage* **23**, 1194-1203, doi:10.1016/j.joca.2015.02.166 (2015).

10 Li, X. C. *et al.* Characteristics and potentials of stem cells derived from human degenerated nucleus pulposus: potential for regeneration of the intervertebral disc. *BMC musculoskeletal disorders* **18**, 242, doi:10.1186/s12891-017-1567-4 (2017).

11 Wang, K. *et al.* The role of angiopoietin-2 in nucleus pulposus cells during human intervertebral disc degeneration. *Laboratory investigation; a journal of technical methods and pathology* **97**, 971-982, doi:10.1038/labinvest.2017.35 (2017).

12 Sakai, D. *et al.* Successful fishing for nucleus pulposus progenitor cells of the intervertebral disc across species. *JOR Spine* **1**, e1018, doi:10.1002/jsp2.1018 (2018).

13 Wu, H. *et al.* Regenerative potential of human nucleus pulposus resident stem/progenitor cells declines with ageing and intervertebral disc degeneration. *Int J Mol Med* **42**, 2193-2202, doi:10.3892/ijmm.2018.3766 (2018).

14 Zeng, X. *et al.* Effect of Conditioned Medium from Human Umbilical Cord-Derived Mesenchymal Stromal Cells on Rejuvenation of Nucleus Pulposus Derived Stem/Progenitor Cells from Degenerated Intervertebral Disc. *Int J Stem Cells* **13**, 257-267, doi:10.15283/ijsc20027 (2020).

15 Zhang, X. *et al.* Spheroid-Like Cultures for Expanding Angiopoietin Receptor-1 (aka. Tie2) Positive Cells from the Human Intervertebral Disc. *International journal of molecular sciences* **21**, doi:10.3390/ijms21249423 (2020).

16 Guerrero, J., Hackel, S., Croft, A. S., Albers, C. E. & Gantenbein, B. The effects of 3D culture on the expansion and maintenance of nucleus pulposus progenitor cell multipotency. *JOR Spine* **4**, e1131, doi:10.1002/jsp2.1131 (2021).

17 Zhang, Y. *et al.* Directed Differentiation of Notochord-like and Nucleus Pulposus-like Cells Using Human Pluripotent Stem Cells. *Cell reports* **30**, 2791-2806 e2795, doi:10.1016/j.celrep.2020.01.100 (2020).

18 Bischof, M. C. *et al.* Influence of Angiopoietin Treatment with Hypoxia and Normoxia on Human Intervertebral Disc Progenitor Cell's Proliferation, Metabolic Activity, and Phenotype. *Applied Sciences* **11**, 7144 (2021).

19 Sako, K. *et al.* Optimization of Spheroid Colony Culture and Cryopreservation of Nucleus Pulposus Cells for the Development of Intervertebral Disc Regenerative Therapeutics. *Applied Sciences* **11**, 3309, doi:10.3390/app11083309 (2021).

20 Sako, K. *et al.* Effect of Whole Tissue Culture and Basic Fibroblast Growth Factor on Maintenance of Tie2 Molecule Expression in Human Nucleus Pulposus Cells. *International journal of molecular sciences* **22**, doi:10.3390/ijms22094723 (2021).

21 Soma, H. *et al.* Recombinant Laminin-511 Fragment (iMatrix-511) Coating Supports Maintenance of Human Nucleus Pulposus Progenitor Cells In Vitro. *International journal of molecular sciences* **24**, doi:10.3390/ijms242316713 (2023).

22 Munesada, D. *et al.* Investigation of the Mitigation of DMSO-Induced Cytotoxicity by Hyaluronic Acid following Cryopreservation of Human Nucleus Pulposus Cells. *International journal of molecular sciences* **24**, doi:10.3390/ijms241512289 (2023).

23 Xia, K. S. *et al.* An esterase-responsive ibuprofen nano-micelle pre-modified embryo derived nucleus pulposus progenitor cells promote the regeneration of intervertebral disc degeneration. *Bioact Mater* **21**, 69-85, doi:10.1016/j.bioactmat.2022.07.024 (2023).

24 Tamagawa, S. *et al.* SOD2 orchestrates redox homeostasis in intervertebral discs: A novel insight into oxidative stress-mediated degeneration and therapeutic potential. *Redox Biol* **71**, 103091, doi:10.1016/j.redox.2024.103091 (2024).

25 Otani, Y. *et al.* Assessment of Tie2-Rejuvenated Nucleus Pulposus Cell Transplants from Young and Old Patient Sources Demonstrates That Age Still Matters. *International journal of molecular sciences* **25**, doi:10.3390/ijms25158335 (2024).

26 Zhang, Y. *et al.* Dedifferentiation-like reprogramming of degenerative nucleus pulposus cells into notochordal-like cells by defined factors. *Molecular therapy : the journal of the American Society of Gene Therapy* **32**, 2563-2583, doi:10.1016/j.ymthe.2024.06.018 (2024).

27 Ogasawara, S. *et al.* Alginate vs. Hyaluronic Acid as Carriers for Nucleus Pulposus Cells: A Study on Regenerative Outcomes in Disc Degeneration. *Cells* **13**, 1984, doi:10.3390/cells13231984 (2024).

28 Ionescu, A. M. *et al.* CD24 Positive Nucleus Pulposus Cells in Adult Human Intervertebral Discs Maintain a More Notochordal Phenotype Than GD2 Positive Cells. *JOR Spine* **7**, e70029, doi:10.1002/jsp2.70029 (2024).
