# Supplementary material for: TIE2-positive cells in the nucleus pulposus with a purpose: the who, what and why
Source: J Biomed Sci. 2026 Mar 2;33:24. doi: 10.1186/s12929-026-01220-7 (PMC12952123; doi:10.1186/s12929-026-01220-7)
Supplement: Supplementary file 4 — Additional file 4. [file 12929_2026_1220_MOESM4_ESM.pdf]

Supplemental data

Supplemental item 4. Overview of Primer Design That Were Employed to Detect *TEK* Expression. The designs were assessed through BLAST to reveal the general specificity and significance relating to the detection of *TEK*.

Authors	Year	Ref	Species	Type	Sequence	BLAST® main hit(s)	Max score	*TEK* detected ?
Tekari et al.	2016	1	Bovine	FW	GGA CAG GCA ATA AGG ATA CG	TEK receptor tyrosine kinase (TEK), mRNA	40.1	✓
				RV	ACC GAG TGG ATG AAG GAA	TEK receptor tyrosine kinase (TEK), mRNA	36.2	
Wu et al.	2018	2	Human	FW	AAT CAC TAT GAG GCT TGG CAA CAT	TEK receptor tyrosine kinase (TEK), transcript variant 1, 2, 3, 4, and 5 mRNA	48.1	✓
				RV	GCG TCT CAC AGG TCC AGG AT	TEK receptor tyrosine kinase (TEK), transcript variant 1, 2, 3, 4, and 5 mRNA	40.1	
Zeng et al.	2020	3	Human	FW	AAT CAC TAT GAG GCT TGG CAA CAT	TEK receptor tyrosine kinase (TEK), transcript variant 1, 2, 3, 4, and 5 mRNA	48.1	✓
				RV	GCG TCT CAC AGG TCC AGG AT	TEK receptor tyrosine kinase (TEK), transcript variant 1, 2, 3, 4, and 5 mRNA	40.1	
Zhang et al.	2020	4	Human	FW	TTA GCC AGC TTA GTT CTC TGT GG	TEK receptor tyrosine kinase (TEK), transcript variant 1, 2, 3, 4, and 5 mRNA	46.1	✓
				RV	AGC ATC AGA TAC AAG AGG TAG GG	TEK receptor tyrosine kinase (TEK), transcript variant 1, 2, 4, and 5 mRNA	46.1	
Guerrero et al.	2020	5	Human	FW	TTA GCC AGC TTA GTT CTC TGT GG	TEK receptor tyrosine kinase (TEK), transcript variant 1, 2, 3, 4, and 5 mRNA	46.1	✓
				RV	AGC ATC AGA TAC AAG AGG TAG GG	TEK receptor tyrosine kinase (TEK), transcript variant 1, 2, 4, and 5 mRNA	46.1	
Bischof et al.	2021	6	Human	FW	TTA GCC AGC TTA GTT CTC TGT GG	TEK receptor tyrosine kinase (TEK), transcript variant 1, 2, 3, 4, and 5 mRNA	46.1	✓
				RV	AGC ATC AGA TAC AAG AGG TAG GG	TEK receptor tyrosine kinase (TEK), transcript variant 1, 2, 4, and 5 mRNA	46.1	
Laagland et al.	2022	7	Canine	FW	CAG CTT ACC AGG TGG ACA TTT TTG	TEK receptor tyrosine kinase (TEK), transcript variant X1, X2, X3, and X4, predicted mRNA	48.1	X
						Sodium voltage-gated channel alpha subunit 9 (SCN9A), transcript variant X1, X2, X3, and X4, predicted, mRNAOL	30.2	
				RV	GTC CGC TGG TGC TTG AGA TTT AG	TEK receptor tyrosine kinase (TEK), transcript variant X1, X2, X3, and X4, predicted mRNA	46.1	

						Zinc finger protein 629 (ZNF629), transcript variant X1, X2, X3, X4, X5, X6, and X7, predicted mRNA	32.2	
						Zinc finger protein 275 (ZNF275), predicted mRNA	30.2	
Chen et al.	2024	8	Mouse	FW	GGG CAG TCT GGT ACT TCC AAG CT	*No hits of significant alignment*	-	X
				RV	ATA TCC CCT TGT TCC CTT TCT GC	Angiomotin-like 1 (Amotl1), transcript variant X1, X2, X3, X4, X5, X6, and X8 predicted mRNA	34.2	
Zhang et al.	2024	9	Human	FW	TTA GCC AGC TTA GTT CTC TGT GG	TEK receptor tyrosine kinase (TEK), transcript variant 1, 2, 3, 4, and 5 mRNA	46.1	✓
				RV	AGC ATC AGA TAC AAG AGG TAG GG	TEK receptor tyrosine kinase (TEK), transcript variant 1, 2, 4, and 5 mRNA	46.1	
			Rat	FW	TGC AGA GAA CAA CAT AGG GTC AAG C	TEK receptor tyrosine kinase (TEK), mRNA	34.2	✓
				RV	AGC ATC AGA TAC AAG AGG TAG GG	cytochrome P450, family 26, subfamily b, polypeptide 1 (Cyp26b1), mRNA	34.2	

Supplemental data

REFERENCES

1 Tekari, A., Chan, S. C. W., Sakai, D., Grad, S. & Gantenbein, B. Angiopoietin-1 receptor Tie2 distinguishes multipotent differentiation capability in bovine coccygeal nucleus pulposus cells. *Stem cell research & therapy* **7**, 75, doi:10.1186/s13287-016-0337-9 (2016).

2 Wu, H. *et al.* Regenerative potential of human nucleus pulposus resident stem/progenitor cells declines with ageing and intervertebral disc degeneration. *Int J Mol Med* **42**, 2193-2202, doi:10.3892/ijmm.2018.3766 (2018).

3 Zeng, X. *et al.* Effect of Conditioned Medium from Human Umbilical Cord-Derived Mesenchymal Stromal Cells on Rejuvenation of Nucleus Pulposus Derived Stem/Progenitor Cells from Degenerated Intervertebral Disc. *Int J Stem Cells* **13**, 257-267, doi:10.15283/ijsc20027 (2020).

4 Zhang, X. *et al.* Spheroid-Like Cultures for Expanding Angiopoietin Receptor-1 (aka. Tie2) Positive Cells from the Human Intervertebral Disc. *International journal of molecular sciences* **21**, doi:10.3390/ijms21249423 (2020).

5 Guerrero, J., Hackel, S., Croft, A. S., Albers, C. E. & Gantenbein, B. The effects of 3D culture on the expansion and maintenance of nucleus pulposus progenitor cell multipotency. *JOR Spine* **4**, e1131, doi:10.1002/jsp2.1131 (2021).

6 Bischof, M. C. *et al.* Influence of Angiopoietin Treatment with Hypoxia and Normoxia on Human Intervertebral Disc Progenitor Cell's Proliferation, Metabolic Activity, and Phenotype. *Applied Sciences* **11**, 7144 (2021).

7 Laagland, L. T. *et al.* Hyperosmolar expansion medium improves nucleus pulposus cell phenotype. *JOR Spine* **5**, e1219, doi:10.1002/jsp2.1219 (2022).

8 Chen, Y. *et al.* Characterization of the Nucleus Pulposus Progenitor Cells via Spatial Transcriptomics. *Adv Sci (Weinh)* **11**, e2303752, doi:10.1002/advs.202303752 (2024).

9 Zhang, Y. *et al.* Dedifferentiation-like reprogramming of degenerative nucleus pulposus cells into notochordal-like cells by defined factors. *Molecular therapy : the journal of the American Society of Gene Therapy* **32**, 2563-2583, doi:10.1016/j.ymthe.2024.06.018 (2024).
