# Supplementary material for: TIE2-positive cells in the nucleus pulposus with a purpose: the who, what and why
Source: J Biomed Sci. 2026 Mar 2;33:24. doi: 10.1186/s12929-026-01220-7 (PMC12952123; doi:10.1186/s12929-026-01220-7)
Supplement: Supplementary file 5 — Additional file 5. [file 12929_2026_1220_MOESM5_ESM.pdf]

Supplemental data

Supplemental item 5. Overview of Reports Assessing the Presence of TIE2+ or *TEK*-Expressing Cells in the NP of Different Species.

Species	Authors	Year	Ref	Level	Life stage	Method	Presence	Range (%) *	Comments
**Mouse**	Sakai et al.	2012	1	Coccygeal	Adult	IHC	✓	Not quantified	Colocalized with Ang-1 positive NP cells
	Ishii et al.	2017	2	Coccygeal	Juvenile	CFA	✓	Not quantified	CFA used as an indirect indicator of Tie2+ NPPCs
	Gao et al.	2022	3	Lumbar & coccygeal	Juvenile	FACS	✓	Not quantified	Positivity not directly reported, but ~64% of Ust2r+ cells were determined Tie2+
	Xia et al.	2023	4	Notochord	Embryo	FACS	✓	~57%	FACS of Tie2-Gfp reporter genetic mouse model
	Tan et al.	2024	5	Lumbar & Coccygeal	Embryo	scRNA-S	✗	0%	
					Juvenile	scRNA-S	✗	0%	
	Chen et al.	2024	6	Notochord	Embryo	ST	✗	0%	
				Unspecified	Juvenile	ST	✗	0%	
						Cell track	✗	0%	
**Rat**	He et al.	2021	7	Lumbar	Juvenile	IHC	✓	~13%	Tie2 positivity rate goes down with age
					Adult	IHC	✓	~4%	
				Coccygeal	Juvenile	IHC	✓	~5% – 8%	Tie2 positivity rate goes down with compression induced disc degeneration
	Zhang et al.	2024	8	Coccygeal	Adult	IHC	✓	~1%	Detection was done in a model of induced disc degeneration and found general low Tie2 positivity rates.
							✓	~10%	The viral mediated overexpression of *Oct4*, *Tbxt*, and *Foxa2*, promoting notochordal cells differentiation resulted in enhanced Tie2 positivity compared to the none treated controls, the enhanced Tie2-positivity associated with an increase in Nog, Shh, Sall3, and Krt8 positive cells as well as reduced apoptosis and Ki67+ cells.
						PCR	✓	N/A	*Tek* expression was detected.
							✓	N/A	The viral mediated overexpression of *Oct4*, *Tbxt*, and *Foxa2* in disc degeneration model, could significantly increase the expression of *Tek* after 4 weeks. This was paired with an increase in Nog, Shh, Sall3, and Krt8 expression.
	Xue et al.	2024	9	Lumbar	Juvenile	scRNA-S	✓	N/A	*Tek* and *B4galnt* (Gd2-gene) were identified in NPSCs in the NP, with double positive cells representing 1.5% of the NP populations
						IHC	✓	Not quantified	Both Tie2 and Gd2 were found in NPSCs in the NP region as well as native stem cell niches
**Pig**	Williams et al.	2023	10	Unspecified	Unspecified	IHC	✓	Not quantified	
**Canine**	Sakai et al.	2018	11	Notochord	Embryo	IHC	✓	0% – 80%	
**Sheep**	Frapin et al.	2020	12	Lumbar	Adult	IHC	✓	Not quantified	CCL5/GDF5/TGF-β1 with ASC homing could enhance TIE2-positivity.
**Bovine**	Tekari et al.	2016	13	Coccygeal	Juvenile	FCM	✓	~9%	
	Frauchiger et al.	2018	14	Coccygeal	Juvenile	FACS	✓	~0% – 13%	Additionally, MACS and Pluriselect methods were also applied and confirmed TIE2+ NP cells, but were suboptimal compared to FACS methods
	Wangler et al.	2019	15	Juvenile	Unspecified	FCM	✓	2% – 4%	Results from ex vivo IVD cultures. Tie2-positivity could be enhanced by MSC homing into the explants.
	Molinos et al.	2023	16	Coccygeal	Juvenile	FCM	✓	~89%	

Supplemental data

					Adult	FCM	✓	~0%	
**Human**	Sakai et al.	2012	1	Lumbar	Adult	IHC	✓	Not quantified	Colocalized with ANG-1 positive NP cells
						FCM	✓	0% – ~85%	Highly dependent on age and degeneration status
	Rodrigues-Pinto et al.	2016	17	Notochord	Embryo	IHC	✗	0%	Report does not present the TIE2-stained samples, nor does it present positive/negative controls confirming the specificity of the antibody employed.
	Sakai et al.	2018	11	Notochord	Embryo to infant	IHC	✓	0% – ~62%	
	Wangler et al.	2019	15	Lumbar	Adult	FCM	✓	0% – ~2%	Results from ex vivo IVD cultures. TIE2-positivity could be enhanced by MSC homing into the explants.
	He et al.	2021	7	Lumbar	Adult	IHC	✓	7% – 34%	TIE2 positivity in Pfirrmann grade II samples was clearly higher than Pfirrmann grade IV samples
	Tan et al.	2024	5	Lumbar	Adult	scRNA-S	✗	0%	Included samples from old patients with degenerative discs
	Ionescu et al.	2024	18	Unspecified	Juvenile to adult	IHC	✓	1.2% – 30.1%	TIE2 positivity remained relatively stable across different degeneration grades.<br>When categorized by age, TIE2 expression showed consistent levels from young AIS patients to adults with disc degeneration, with a slight but significant increase observed in seniors (65–75 years).

* Values presented with a ~ symbol are estimated values taken from graphs included in the article. Abbreviations: CFA – Colony forming assay, FACS – Fluorescence activated cell sorting, FCM – Flow cytometry, IHC – Immunohistochemistry, IVD – Intervertebral disc, MACS – Magnetic activated cell sorting, MSC – Mesenchymal stromal cells, N/A – Not applicable, NP – Nucleus pulposus, scRNA-S – Single cell RNA sequencing, ST – Spatialranscriptomics

Supplemental data

REFERENCES

1 Sakai, D. *et al.* Exhaustion of nucleus pulposus progenitor cells with ageing and degeneration of the intervertebral disc. *Nat Commun* **3**, 1264, doi:10.1038/ncomms2226 (2012).

2 Ishii, T. *et al.* Sciatic nerve regeneration by transplantation of in vitro differentiated nucleus pulposus progenitor cells. *Regen Med* **12**, 365-376, doi:10.2217/rme-2016-0168 (2017).

3 Gao, B. *et al.* Discovery and Application of Postnatal Nucleus Pulposus Progenitors Essential for Intervertebral Disc Homeostasis and Degeneration. *Adv Sci (Weinh)* **9**, e2104888, doi:10.1002/advs.202104888 (2022).

4 Xia, K. S. *et al.* An esterase-responsive ibuprofen nano-micelle pre-modified embryo derived nucleus pulposus progenitor cells promote the regeneration of intervertebral disc degeneration. *Bioact Mater* **21**, 69-85, doi:10.1016/j.bioactmat.2022.07.024 (2023).

5 Tan, Z. *et al.* Progenitor-like cells contributing to cellular heterogeneity in the nucleus pulposus are lost in intervertebral disc degeneration. *Cell reports* **43**, 114342, doi:10.1016/j.celrep.2024.114342 (2024).

6 Chen, Y. *et al.* Characterization of the Nucleus Pulposus Progenitor Cells via Spatial Transcriptomics. *Adv Sci (Weinh)* **11**, e2303752, doi:10.1002/advs.202303752 (2024).

7 He, R. *et al.* HIF1A Alleviates compression-induced apoptosis of nucleus pulposus derived stem cells via upregulating autophagy. *Autophagy* **17**, 3338-3360, doi:10.1080/15548627.2021.1872227 (2021).

8 Zhang, Y. *et al.* Dedifferentiation-like reprogramming of degenerative nucleus pulposus cells into notochordal-like cells by defined factors. *Molecular therapy : the journal of the American Society of Gene Therapy* **32**, 2563-2583, doi:10.1016/j.ymthe.2024.06.018 (2024).

9 Xue, B. *et al.* A Novel Superparamagnetic-Responsive Hydrogel Facilitates Disc Regeneration by Orchestrating Cell Recruitment, Proliferation, and Differentiation within Hostile Inflammatory Niche. *Adv Sci (Weinh)* **11**, e2408093, doi:10.1002/advs.202408093 (2024).

10 Williams, R. J. *et al.* Recommendations for intervertebral disc notochordal cell investigation: From isolation to characterization. *JOR Spine* **6**, e1272, doi:10.1002/jsp2.1272 (2023).

11 Sakai, D. *et al.* Successful fishing for nucleus pulposus progenitor cells of the intervertebral disc across species. *JOR Spine* **1**, e1018, doi:10.1002/jsp2.1018 (2018).

12 Frapin, L. *et al.* Controlled release of biological factors for endogenous progenitor cell migration and intervertebral disc extracellular matrix remodelling. *Biomaterials* **253**, 120107, doi:10.1016/j.biomaterials.2020.120107 (2020).

13 Tekari, A., Chan, S. C. W., Sakai, D., Grad, S. & Gantenbein, B. Angiopoietin-1 receptor Tie2 distinguishes multipotent differentiation capability in bovine coccygeal nucleus pulposus cells. *Stem cell research & therapy* **7**, 75, doi:10.1186/s13287-016-0337-9 (2016).

14 Frauchiger, D. A. *et al.* Fluorescence-Activated Cell Sorting Is More Potent to Fish Intervertebral Disk Progenitor Cells Than Magnetic and Beads-Based Methods. *Tissue Eng Part C Methods* **25**, 571-580, doi:10.1089/ten.TEC.2018.0375 (2019).

15 Wangler, S. *et al.* Mesenchymal Stem Cell Homing Into Intervertebral Discs Enhances the Tie2-positive Progenitor Cell Population, Prevents Cell Death, and Induces a Proliferative Response. *Spine (Phila Pa 1976)* **44**, 1613-1622, doi:10.1097/BRS.0000000000003150 (2019).

16 Molinos, M. *et al.* Alterations of bovine nucleus pulposus cells with aging. *Aging Cell* **22**, e13873, doi:10.1111/acel.13873 (2023).

17 Rodrigues-Pinto, R. *et al.* Spatiotemporal analysis of putative notochordal cell markers reveals CD24 and keratins 8, 18, and 19 as notochord-specific markers during early human intervertebral disc development. *J Orthop Res* **34**, 1327-1340, doi:10.1002/jor.23205 (2016).

18 Ionescu, A. M. *et al.* CD24 Positive Nucleus Pulposus Cells in Adult Human Intervertebral Discs Maintain a More Notochordal Phenotype Than GD2 Positive Cells. *JOR Spine* **7**, e70029, doi:10.1002/jsp2.70029 (2024).
