# Supplementary material for: TIE2-positive cells in the nucleus pulposus with a purpose: the who, what and why
Source: J Biomed Sci. 2026 Mar 2;33:24. doi: 10.1186/s12929-026-01220-7 (PMC12952123; doi:10.1186/s12929-026-01220-7)
Supplement: Supplementary file 6 — Additional file 6. [file 12929_2026_1220_MOESM6_ESM.pdf]

Supplemental data

Supplemental item 6. Tabular Overview of TIE2-Positivity in Human Notochordal Tissue. This table presents TIE2-positivity rates in human fetal and infant nucleus pulposus (NP) tissues across various gestational and postnatal ages. The data are derived from previously reported samples in Sakai et al. [1], as referenced in their Supplemental Figure 1. Prenatal ages are expressed as weeks of gestation, while post-natal ages are given as time elapsed since birth. The "Cell types" column indicates the types of cells identified within the NP tissue. The "NCC" and "cNPC" columns specify whether notochordal cells (NCC) or fibrocartilage NP cells (cNPC) were found to be TIE2+.

No	Age	Pre/Post-natal	TIE2+ cell	Cell count	TIE2-positivity	Cell types	NCC	cNPC
1	20 weeks	Pre-natal	0	-	0%	-	-	-
2	21 weeks	Pre-natal	0	-	0%	-	-	-
3	21 weeks	Pre-natal	0	-	0%	-	-	-
4	21 weeks	Pre-natal	9	77	12%	cNPC & NCC	V	X
5	22 weeks	Pre-natal	0	-	0%	-	-	-
6	22 weeks	Pre-natal	57	184	31%	cNPC	X	V
7	23 weeks	Pre-natal	0	-	0%	-	-	-
8	26 weeks	Pre-natal	16	45	36%	cNPC	X	V
9	28 weeks	Pre-natal	0	-	0%	-	-	-
10	2 days	Post-natal	50	213	23%	cNPC & NCC	V	V
11	3 days	Post-natal	9	19	47%	cNPC	X	V
12	5 days	Post-natal	20	42	48%	cNPC & NCC	V	V
13	6 days	Post-natal	8	26	31%	cNPC	X	V
14	2.5 weeks	Post-natal	0	-	0%	-	-	-
15	3 months	Post-natal	18	28	64%	cNPC & NCC	V	V

REFERENCES

1 Sakai, D. *et al.* Successful fishing for nucleus pulposus progenitor cells of the intervertebral disc across species. *JOR Spine* **1**, e1018, doi:10.1002/jsp2.1018 (2018).
