# Supplementary material for: TIE2-positive cells in the nucleus pulposus with a purpose: the who, what and why
Source: J Biomed Sci. 2026 Mar 2;33:24. doi: 10.1186/s12929-026-01220-7 (PMC12952123; doi:10.1186/s12929-026-01220-7)
Supplement: Supplementary file 7 — Additional file 7. [file 12929_2026_1220_MOESM7_ESM.pdf]

Supplemental data

Supplemental item 7. Graphical Overview of TIE2-Positivity in Human Notochordal Cell-Rich Tissue. Complementing Supplemental Item 2, this figure illustrates preliminary assessment of differences in TIE2-positivity across previously reported human fetal and infant NP tissues, as published in Sakai et al. [1] and referenced in their Supplemental Figure 1. (A) Bar graphs comparing TIE2-positivity rates between pre- and postnatal NP tissues. Data normality was evaluated using the Shapiro-Wilk test, which indicated a nonparametric distribution; therefore, statistical significance was assessed using the Mann-Whitney test. (B) Scatter plot of TIE2-positivity rates relative to tissue age (assuming a gestational age of 40 weeks at birth), revealing a weak positive linear correlation between developmental age and the presence of TIE2-positive cells. Note that caution is advised in interpreting these findings, as they are based on a small sample set, the cause of abortion is unknown, and processing conditions may not have been entirely uniform across all samples. Moreover, these data plots are original, previously unpublished, data used solely as supporting observations to trend identified from the literature. *Abbreviation: TIE2 – Tyrosine kinase with immunoglobulin-like.*

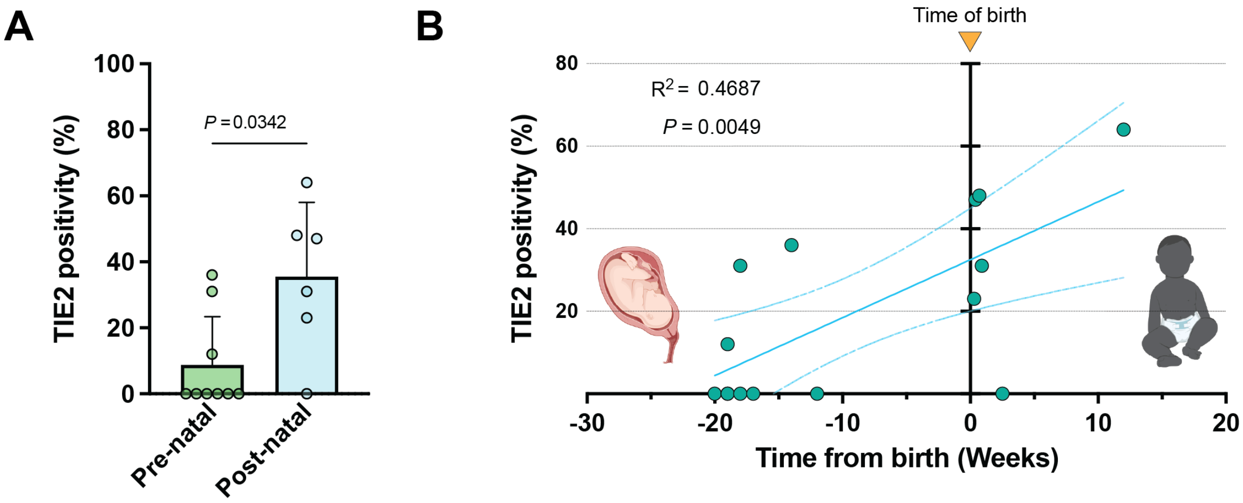

REFERENCES

1 Sakai, D. *et al.* Successful fishing for nucleus pulposus progenitor cells of the intervertebral disc across species. *JOR Spine* **1**, e1018, doi:10.1002/jsp2.1018 (2018).
