# Supplementary material for: TIE2-positive cells in the nucleus pulposus with a purpose: the who, what and why
Source: J Biomed Sci. 2026 Mar 2;33:24. doi: 10.1186/s12929-026-01220-7 (PMC12952123; doi:10.1186/s12929-026-01220-7)
Supplement: Supplementary file 8 — Additional file 8. [file 12929_2026_1220_MOESM8_ESM.pdf]

Supplemental data

Supplemental item 8. Isotype controls dog-stained sections. Isotype controls matching the canine stained specimen shown in figure 5, confirming the specificity of the TIE2 staining. [1] Note, the bottom left panel under "notochordal cell-retaining species" shows tissue from a client-owned Golden Retriever (10 years old) presenting with severe disc degeneration and low back pain in predilection sites (cervical and lower lumbar regions, similar to those affected in human IVD disease). Although non-chondrodystrophic by breed, this dog had lost the predominance of notochordal cells in the NP tissue as part of the degenerative process. Note: Images include original observational data provided to illustrate and support trends described in the literature. Abbreviations: *IDD – Intervertebral disc degeneration.*

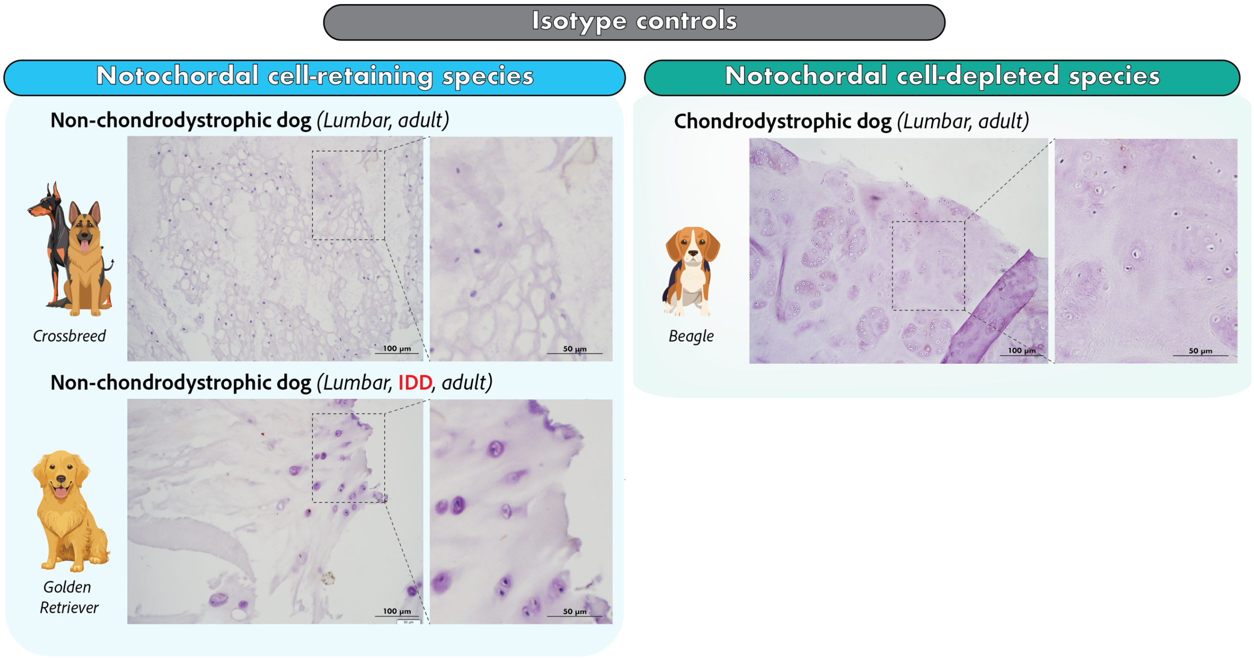

REFERENCES

1 Laagland, L. T. *et al.* Hyperosmolar expansion medium improves nucleus pulposus cell phenotype. *JOR Spine* **5**, e1219, doi:10.1002/jsp2.1219 (2022).
